# Sunda arc mantle source δ^18^O value revealed by intracrystal isotope analysis

**DOI:** 10.1038/s41467-021-24143-3

**Published:** 2021-06-24

**Authors:** Frances M. Deegan, Martin J. Whitehouse, Valentin R. Troll, Harri Geiger, Heejin Jeon, Petrus le Roux, Chris Harris, Marcel van Helden, Osvaldo González-Maurel

**Affiliations:** 1 Department of Earth Sciences, Natural Resources and Sustainable Development (NRHU), Uppsala University, Uppsala, Sweden; 2 Department of Geosciences, Swedish Museum of Natural History, Stockholm, Sweden; 3 Faculty of Geological Engineering, Universitas Padjajaran (UNPAD), Bandung, Indonesia; 4 Institute of Earth and Environmental Sciences, University of Freiburg, Freiburg, im Breisgau Germany; 5 Department of Geological Sciences, University of Cape Town, Cape Town, South Africa; 6 Department of Earth Sciences, Vrije Universiteit Amsterdam, Amsterdam, Netherlands

**Keywords:** Geochemistry, Petrology, Volcanology

## Abstract

Magma plumbing systems underlying subduction zone volcanoes extend from the mantle through the overlying crust and facilitate protracted fractional crystallisation, assimilation, and mixing, which frequently obscures a clear view of mantle source compositions. In order to see through this crustal noise, we present intracrystal Secondary Ion Mass Spectrometry (SIMS) δ^18^O values in clinopyroxene from Merapi, Kelut, Batur, and Agung volcanoes in the Sunda arc, Indonesia, under which the thickness of the crust decreases from ca. 30 km at Merapi to ≤20 km at Agung. Here we show that mean clinopyroxene δ^18^O values decrease concomitantly with crustal thickness and that lavas from Agung possess mantle-like He-Sr-Nd-Pb isotope ratios and clinopyroxene mean equilibrium melt δ^18^O values of 5.7 ‰ (±0.2 1 SD) indistinguishable from the δ^18^O range for Mid Ocean Ridge Basalt (MORB). The oxygen isotope composition of the mantle underlying the East Sunda Arc is therefore largely unaffected by subduction-driven metasomatism and may thus represent a sediment-poor arc end-member.

## Abstract (web summary)

Subduction zone volcanoes are underlain by extensive magma plumbing systems, which can obscure original mantle source signals. Here, the authors show that intra-crystal oxygen isotope analysis of clinopyroxenes from the Sunda arc (Indonesia) reveal the δ18 O value of the sub-arc mantle.

## Introduction

**Fig. 1:**
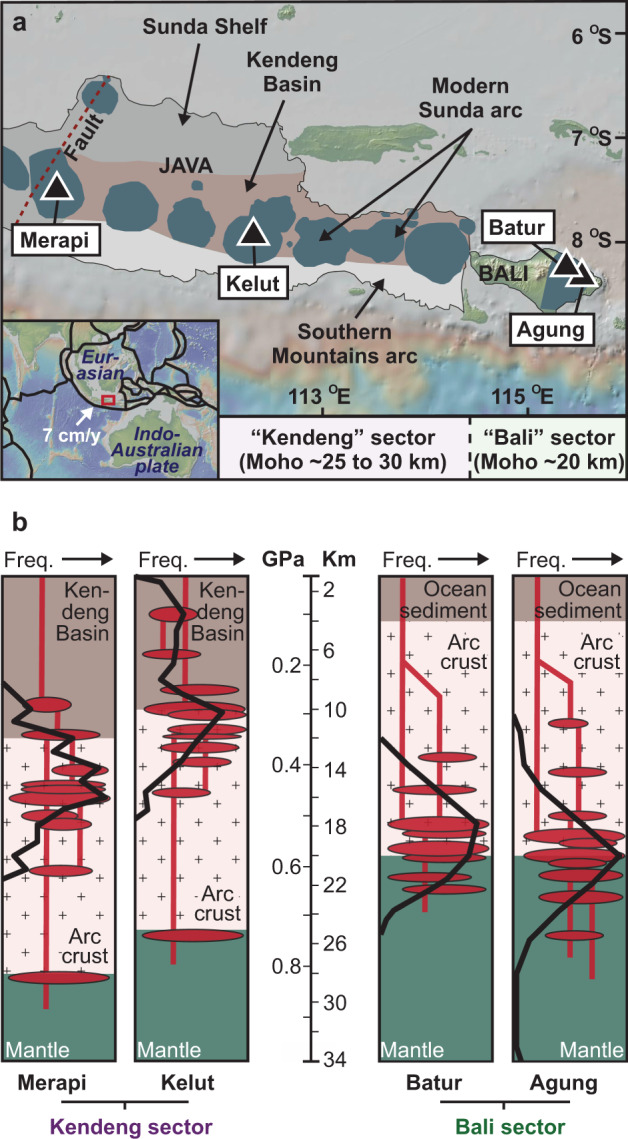
Study area and magma storage in Java and Bali. **a** Map of Central to East Java and Bali showing the location of Merapi, Kelut, Batur, and Agung volcanoes. The modern Sunda arc volcanoes in Central to East Java (dark grey) are mainly built on the Kendeng Basin (brown), but overlap locally with the Miocene Southern Mountains arc. Inset shows the regional tectonic setting with the study area in a red box. Maps in **a** were produced using GeoMapApp (www.geomapapp.org)^74^ with geological information from ref. ^59^. **b** Summary of thermobarometric models constructed using clinopyroxene mineral data from the samples utilised in this study. Black curves represent the frequency of crystallisation depths calculated from clinopyroxene mineral data (data from refs. ^12–14^). Note that clinopyroxene from the Merapi and Kelut samples crystallised dominantly in the arc crust (including the Kendeng Basin), whereas clinopyroxene from the Balinese samples crystallised at the crust–mantle boundary and within the upper mantle. Abbreviations: Freq., frequency; Moho, Mohorovičić discontinuity.

Magma storage beneath subduction zone (arc) volcanoes typically comprises a series of interconnected magma bodies that extend vertically from the mantle to the surface, spanning the entire crustal column (e.g. refs. ^1–3^). A prime example of this phenomenon is the Sunda arc in Indonesia (Fig. 1), where abundant geophysical and petrological evidence for lithologically controlled magma storage horizons has been documented in regional studies of the Sunda arc^4,5^ and at specific volcanoes such as Krakatau, Sunda Strait^6^; Merapi, Central Java^7–12^; Kelut, East Java^13^; and Agung and Batur, Bali^14^. When magma is stored in the crust prior to eruption, its original geochemical characteristics are usually modified by fractional crystallisation, crustal assimilation, and mixing with isotopically diverse melts and fluids (e.g.^15–20^). These processes can obscure the primary mantle sources of arc volcanism^16,21^ and lead to considerable uncertainty surrounding the compositions of primary arc melts that are produced in the mantle wedge due to subduction.

**Fig. 2:**
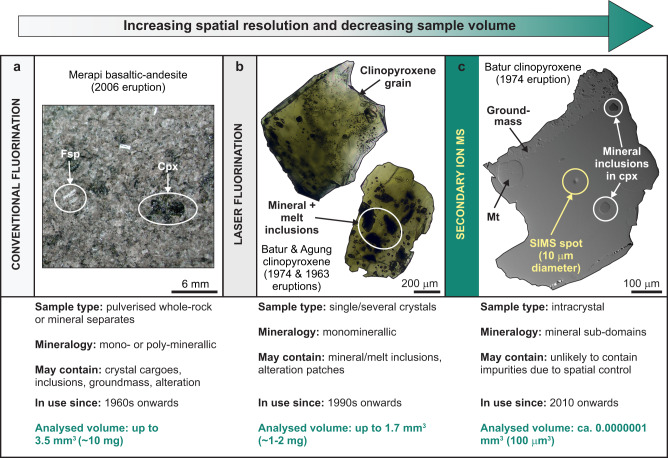
Evolving approaches to oxygen isotope analysis. **a** Photograph of a lava sample from Merapi with visible feldspar and clinopyroxene phenocrysts. Conventional fluorination (CF) is typically utilised to analyse oxygen isotope ratios of pulverised bulk lava and/or mineral separates. **b** Extended focus images (EFI) of polished clinopyroxene crystals in Agung and Batur lavas showing internal features such as mineral and melt inclusions. Laser fluorination (LF) is typically utilised to analyse oxygen isotopes in single minerals or mineral separates. **c** Back Scattered Electron (BSE) image of a clinopyroxene mineral grain from Batur in topographic mode showing surface features. Although this grain also contains mineral inclusions exposed at the polished surface, these are easily avoided with SIMS due to the high degree of spatial control. Abbreviations: Cpx, clinopyroxene; Fsp, feldspar; MS, mass spectrometry; Mt, magnetite.

Oxygen isotopes ratios (^18^O/^16^O reported in standard δ-notation relative to Vienna Standard Mean Ocean Water, i.e., δ^18^O) are frequently employed to study magma genesis and evolution at arcs (e.g.^12,20,22–32^). Initially, our knowledge of the δ^18^O values of subduction zone lavas relied on analysis of pulverised whole-rocks or mineral separates of ca. 3.5 mm^3^ sample volume (~10 mg) per analysis (e.g.^25^), but drawbacks include that whole-rocks are prone to groundmass contamination and/or alteration, and their δ^18^O values represent weighted averages of their mineral and groundmass constituents^16^ (Fig. 2a). Analysis of single mineral grains of up to 1.7 mm^3^ sample volume (~1–2 mg) by Laser Fluorination (LF) largely circumvented these problems and saw a significant improvement in our ability to gain insight into the oxygen isotope compositions of arc systems (e.g.^20,22,23,27–30,32^). Limitations to this approach, however, include that mineral grains often contain inclusions or are partially altered, possessing contrasting δ^18^O values to the host mineral, which may bias the δ^18^O value of the total analysed material (e.g. ref. ^20^; Fig. 2b).

How, then, should we accurately determine mineral δ^18^O values to distinguish crustal from mantle compositions at arc volcanoes? In this work we demonstrate a way forward that involves utilising intracrystal oxygen isotope analysis of phenocrysts in arc lavas, which can be achieved with Secondary Ion Mass Spectrometry (SIMS). The most recent generation of multicollector, large-radius SIMS instruments offer a typical spatial resolution of <10 µm and sample at <1 µm depth (equivalent to a minute sample volume of just 0.0000001 mm^3^ or 100 µm^3^ of material per analysis), which permits a high degree of spatial control and analysis of unadulterated, inclusion-free domains within individual crystals^33^ (Fig. 2c). However, SIMS analysis of major igneous phenocryst phases has thus far been relatively untapped for volcanic arcs (exceptions include quartz^34^ from Toba volcano and plagioclase^35^ from Merapi volcano, both in the Sunda arc), due to issues relating to the paucity of well-characterised reference materials, which only recently became available for augitic clinopyroxene^12^. Consequently, there now exists an opportunity to see through the arc and constrain primitive, sub-arc mantle oxygen isotope compositions by approaching this problem in a large-scale, systematic fashion, which we have undertaken here by utilising recently erupted, well-characterised materials from the Sunda arc in Indonesia.

## Results and discussion

### Magma plumbing in the Sunda arc

Early-grown mafic mineral phases such as olivine and pyroxene hold key information on both the architecture of deep magma plumbing systems and on the composition of melts feeding the overlying volcanoes (e.g.^20^). Most of the recent volcanic products of the Sunda arc are typical subduction zone basaltic-andesites (ca. 50 to 57 wt.% SiO_2_) and contain relatively scant olivine (cf.^10,13,14,30,36–38^). Clinopyroxene, on the other hand, is a ubiquitous mineral phase in Sunda arc lavas with the potential to provide valuable insights into source compositions and subsequent magmatic evolution owing to its wide crystallisation pressure and its refractory nature with respect to oxygen isotope diffusional exchange^22^. In this contribution, we present 298 SIMS intracrystal δ^18^O values from clinopyroxene phenocrysts from four volcanoes forming a partial transect of ca. 550 km length of the Sunda arc in Indonesia, comprising Merapi in Central Java, Kelut in East Java, and Batur and Agung on Bali (Fig. 1).

The Sunda arc is a ca. 5600 km long, mixed oceanic-continental arc system generated by subduction of the Indo-Australian plate beneath the Eurasian plate. From Central- to East Java to the islands east of Bali (Lombok, Sumbawa, Flores, and Timor), subduction occurs beneath progressively thinning crust, ranging from ca. 30 km underneath Merapi in Central Java to ≤20 km under Batur and Agung on Bali and further east^39–41^. Although the amount of sediment available for subduction along the Sunda arc varies in terms of thickness and type over length scales of 1000’s of kilometres^42^, subducted sediments are at their minimum thickness offshore East Java and Bali (200–400 m thick^42,43^) and sedimentary accretionary trench infill is virtually absent in this region^44^. The regional tectonics therefore increase the likelihood of obtaining a robust estimate of primitive sub-arc mantle δ^18^O values by examining erupted products from Batur and Agung on Bali. By the same token, we can test for crustal overprinting of primitive δ^18^O values by utilising the erupted products from Merapi and Kelut, where this effect is hypothesised to be stronger due to the greater crustal thickness and the presence of the Kendeng sedimentary basin in the upper crust under these volcanoes, which can promote differentiation and crustal contamination of primary arc melts^45^.

All four of the volcanoes selected for this study have displayed recent eruptive activity and their lavas contain fresh, augitic clinopyroxene phenocrysts (Fig. 2 and Supplementary Fig. 1), whose mineral chemistry has previously been used to establish the depths of the main magma storage reservoirs feeding the overlying volcanoes^21^. Thermobarometric studies have shown that clinopyroxene in both Merapi’s 2006 lava and Kelut’s 2007 dome-forming lava formed in the mid- to upper- arc crust^12,13^, while clinopyroxene in the 1974 lava at Batur and the 1963 lava at Agung formed at the crust-mantle boundary and within the upper mantle beneath Bali^14^ (Fig. 1b). In this study, we present δ^18^O values for clinopyroxene phenocrysts from lavas from each of these recent eruptions, all obtained at the NordSIMS ionprobe facility in Stockholm, Sweden (*n* = 298 analyses in total; see Methods and Supplementary Table 1). In addition, a number of complementary LF (single mineral δ^18^O values) and radiogenic isotope data (host lava Sr–Nd–Pb isotopes) were obtained at the University of Cape Town, South Africa (Supplementary Tables 2, 3). Mineral chemistry and SIMS data are provided in Supplementary Tables 4, 5.

### Clinopyroxene as a probe of primitive arc δ^18^O values

**Fig. 3:**
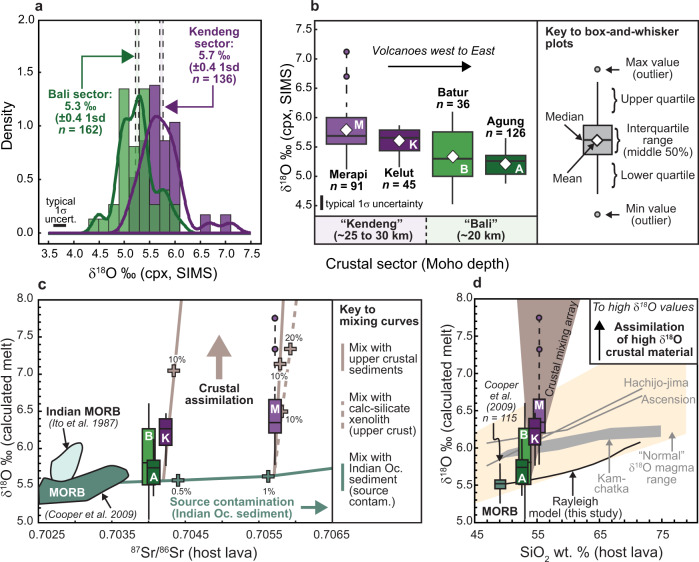
Oxygen isotope results and models. **a** Histograms with density plot overlays showing grain averaged clinopyroxene SIMS δ^18^O values for investigated volcanoes from the Bali and Kendeng sectors. Histogram bins are at 0.2 ‰. **b** Box-and-whisker plots showing grain averaged clinopyroxene SIMS δ^18^O values for each studied volcano. White diamond symbols denote the overall mean δ^18^O clinopyroxene value for each data group. **c** Box-and-whisker plots showing equilibrium melt δ^18^O values calculated from the SIMS data for Agung (A), Batur (B), Kelut (K), and Merapi (M) versus ^87^Sr/^86^Sr in host lavas. Source and crustal contamination models are shown utilising average MORB from refs. ^48,49^, Indian Ocean sediment after ref. ^29^, and Java crustal compositions in refs. ^13,31,35^. **d** Equilibrium melt δ^18^O values versus SiO_2_ contents of host lavas. Oxygen isotope fractionation trajectories are shown in grey for several natural systems after refs. ^24,75^. A closed system Rayleigh fractionation model was additionally calculated based on ref. ^18^ starting from mean MORB^48^. Oxygen isotope magma–crust mixing trajectories are shown in brown, utilising Java crustal compositions in refs. ^13,31,35^. Average propagated uncertainties are 0.25‰ (1σ) for the SIMS data and 0.000011 (2σ) for the Sr isotope data (smaller than symbol size). Abbreviations: cpx, clinopyroxene; Moho, Mohorovičić discontinuity; Oc, ocean; uncert., uncertainty.

We grouped the volcanoes studied here into two crustal sectors with Merapi and Kelut assigned to the “Kendeng” sector, due to their spatial association with the Kendeng sedimentary basin in Central to East Java, and Batur and Agung to the “Bali” sector, due to their location on the thinner crust beneath Bali Island (Fig. 1). The overall mean clinopyroxene δ^18^O value for the Kendeng sector is 5.7 ‰ (±0.4 1sd, *n* = 136) versus 5.3 ‰ (±0.4 1sd, *n* = 162) for the Bali sector (Fig. 3a). All mean clinopyroxene SIMS δ^18^O values reported here overlap with the range for mantle-derived clinopyroxene of 5.6 ± 0.4 ‰^46^ and all show good agreement with the available LF δ^18^O values for similar samples (Supplementary Fig. 2). On a per volcano basis, the mineral grain averaged clinopyroxene δ^18^O values decrease from Central and East Java to Bali: Merapi = 5.8 ‰ (±0.5 1sd, *n* = 91), Kelut = 5.6 ‰ (±0.2 1sd, *n* = 45), Batur = 5.3 ‰ (±0.4 1sd, *n* = 36), Agung = 5.2 ‰ (±0.2 1sd, *n* = 126) (Fig. 3b). This trend of decreasing mean δ^18^O values from west to east (adjusted *R*^2^ = 0.3) mirrors the overall decreasing depth of the Moho from Merapi towards Bali. Furthermore, the difference between the mean δ^18^O value for clinopyroxene from Merapi (westernmost studied volcano) and Agung (easternmost studied volcano) is 0.6 ‰, which is significant considering the uncertainties on the SIMS data (see “Methods”). Our SIMS data thus allow us to capture a previously unobserved arc-wide trend in clinopyroxene δ^18^O values.

Focussing first on the eastern part of the studied arc segment, the Agung lava sample utilised in our SIMS study possesses the most primitive ^206^Pb/^204^Pb, ^207^Pb/^204^Pb, and ^208^Pb/^204^Pb ratios of the four volcanoes studied here, at 18.445 (±0.001; *n* = 2), 15.591 (±0.001; *n* = 2), and 38.526 (±0.003; *n* = 2), respectively (Supplementary Table 2). These ratios overlap with values reported for Indian MORB, placing our Agung sample among the most isotopically primitive erupted products in the entire arc segment (Supplementary Fig. 3). Furthermore, our Agung and Batur lavas have ^87^Sr/^86^Sr and ^143^Nd/^144^Nd ratios that approach the range of values reported for Indian MORB, and their mafic minerals have ^3^He/^4^He ratios that lie within the mantle range of 8 ± 1 R_A_ (ref. ^47^). We therefore argue that clinopyroxene crystals from Bali, and particularly Agung, largely escaped the effects of crustal overprinting and represent robust archives of primitive Sunda Arc oxygen isotope ratios. The mean clinopyroxene SIMS δ^18^O values from both Batur and Agung are, in fact, indistinguishable within analytical uncertainty, at 5.3 and 5.2 ‰. These combined isotopic characteristics point to preservation of mantle isotopic signatures in ferromagnesian minerals from Batur and Agung, consistent with clinopyroxene crystallisation at the crust-mantle boundary beneath Bali.

To gain an insight into the corresponding magmatic δ^18^O values, we calculated equilibrium melt δ^18^O values for i) grain averaged clinopyroxene analysed by SIMS (Supplementary Table 1), and ii) ferromagnesian minerals analysed by LF, including the new data presented here as well as data for similar samples from Merapi, Kelut, Batur, and Agung in the literature (Supplementary Table 3). All melt calculations were performed utilising the SiO_2_-dependent formulations in table 2 of ref. ^24^. For Batur and Agung, clinopyroxene mean δ^18^O values calculate to mean equilibrium melt values of 5.8 (±0.4 1sd, *n* = 36) and 5.7 ‰ (±0.2 1sd, *n* = 126), respectively. Since Agung shows the most primitive radiogenic isotope compositions of all our samples, as well as a tighter range of δ^18^O values than Batur, we argue that Agung represents the most robust estimate of the primary δ^18^O value in the region. This argument is supported by the fact that the calculated melt δ^18^O values for Agung show strong overlap with the range for MORB, which has most recently been assigned a mean value of 5.5 ‰ (±0.1 1sd, *n* = 115)^48^ (note that earlier estimates for MORB were around 5.7 ‰ ±0.3, e.g. refs. ^46,49,50^).

We also present two new LF analyses of olivine grains from Batur, with δ^18^O values of 4.5 and 4.8 ‰. When combined with two existing olivine LF analyses in the literature, the mean δ^18^O value for Batur olivine equates to 4.7 ‰ (±0.2 1sd, *n* = 4). Olivine from mantle xenoliths yield a predominant δ^18^O value of 5.2 to 5.3 ‰ (refs. ^46,51^), which for ultramafic melt equilibrium at 1300°C would suggest melt δ^18^O values of 5.5 to 5.6 ‰. Thus, olivine from Batur possess mineral δ^18^O values lower than olivine equilibrated with ultramafic melt at high temperature. However, this difference is perhaps expected since the δ^18^O values of olivine in our samples are controlled by mineral-melt fractionation in a crystallising basaltic-andesitic magma. Indeed, the δ^18^O values for Batur olivine translate to a mean melt value of 5.8 ‰ (±0.2 1sd, *n* = 4), identical to the mean melt value obtained from the SIMS clinopyroxene data. These results lead us to suggest that the bulk of Batur olivine and clinopyroxene crystallised under equilibrium conditions at the crust-mantle boundary and overall reflect the composition of primitive mantle-derived melts in the region.

In order to constrain the influence of source contamination (subducted sediment input) on δ^18^O values along the studied arc segment, we performed a mixing model in δ^18^O-^87^Sr/^86^Sr space utilising average MORB^48^ and Indian Ocean sediment (“sed A” in ref. ^52^) (Fig. 3c). The model results show that Agung, Batur, and Kelut volcanoes record low degrees of source contamination (less than 0.5 %), while Merapi records ca. 1% contamination, which suggests that the Java-Bali segment of the Sunda arc is relatively sediment-poor. These results are in line with previous site-specific and regional studies that suggest slightly higher portions of sediment being subducted beneath Central Java compared to Bali (e.g. refs. ^29,42–44^). The high δ^18^O values recorded by some fraction of clinopyroxene at all of the studied volcanoes (least and most pronounced at Agung and Merapi, respectively) must therefore arise from crustal contamination, as discussed below. In summary, an important implication of our results is that the mantle source beneath Bali is seemingly not significantly enriched in ^18^O from crustal oxygen via subduction metasomatism, consistent with earlier estimates of as little as 1 or 2% crustal oxygen in the source of most oceanic arc lavas^28^.

### Crustal overprinting of primary δ^18^O values

We now consider the relatively high mean δ^18^O values recorded by clinopyroxene crystals from Merapi and Kelut, which contrast with the lower values for Batur and Agung discussed above. We hypothesise that these overall higher mean δ^18^O values are a function of the composition and thickness of the underlying arc crust, which is continental to transitional under Central Java (ca. 30 km thickness), and thins eastward to become oceanic in character beneath Bali (ca. 20 km thickness)^39–41^, thus facilitating the presence of mafic plumbing systems in the mid- to upper arc crust in Central and Eastern Java^14,21^. The mean clinopyroxene δ^18^O values obtained for Kelut and Merapi calculate to melt δ^18^O values of 6.2 and 6.5 ‰, respectively, which are significantly higher than each of the calculated melt values for the Bali sector (5.8 ‰ on average), Agung volcano (5.7 ‰ on average), and unmodified, MORB-type upper mantle melts (5.5 ‰ on average^48^). The elevated δ^18^O melt values from Central and East Java might reflect the addition of a slab-derived fluid possessing exotic oxygen isotope compositions to the magma source region (cf. ref. ^32^). However, the relatively more evolved compositions of Kelut and Merapi lavas compared to the Balinese lavas (with respect to SiO_2_ content and radiogenic isotope ratios; Fig. 3), coupled with the mid-crustal crystallisation pressures obtained for Kelut and Merapi clinopyroxene, points to magmatic evolution processes in the arc crust, such as fractional crystallisation and crustal assimilation, as the driving force for ^18^O enrichment of some of our Kelut and Merapi clinopyroxene. In fact, at Kelut and Merapi, the mean melt δ^18^O values are 0.4 ‰ and 0.7 ‰ higher than the melt value estimated for the Bali sector, respectively. Yet, numerical fractionation models^24^ show that fractionation of arc basalt (SiO_2_ of 50 wt.%) to basaltic andesite (SiO_2_ of 55 wt.%) under various crystallisation conditions will increase the melt δ^18^O value by <0.2 ‰ (Fig. 3d). Fractional crystallisation is thus unlikely to be the sole reason for the elevated δ^18^O values observed in clinopyroxene, which peak at 7.1 ‰ (equivalent melt δ^18^O value of 7.8 ‰) for a bulk lava SiO_2_ content of 56 wt.% at Merapi.

We therefore posit that the melt in which clinopyroxene with relatively high δ^18^O values grew was contaminated in the mid- to upper crust by crustal material enriched in ^18^O, such as carbonates, calc-silicates and/or siliciclastic sedimentary rocks derived from sedimentary basin fill and altered volcanic debris, which are all present to variable degrees under Central and East Java^13,31,53^. Moreover, recently erupted lavas at Kelut and Merapi contain a variety of crustal xenoliths, including some of siliciclastic and carbonate sedimentary origin, which provide first-hand evidence for magma–crust interaction in the region (e.g. refs. ^13,31,53^). The Sr isotope ratios in lavas from Central and East Java are consistent with this model, as the Merapi and Kelut lavas utilised in this study have mean ^87^Sr/^86^Sr ratios of 0.705716 ± 0.000010 and 0.704244 ± 0.000010, respectively, which extend beyond the likely ^87^Sr/^86^Sr ratios of both uncontaminated arc volcanics^54^ and lavas from Batur and Agung. Indeed, the Sr isotope composition of the Merapi 2006 lava samples overlap regional crustal values and are similar to data reported in previous studies that argue for extensive magma–crust interaction at Merapi^53,55^. Furthermore, helium isotopes in Merapi fumarole gases range from 5.4 to 5.7 R_A_ (ref. ^47^) and are distinct from the mantle-like R_A_ values reported for minerals from Batur and Agung (7.4 to 8.8 R_A_; refs. ^14,47,56^), suggesting that Merapi magma interacted with crustal material with characteristically low ^3^He/^4^He ratios (see also ref. ^31^). By employing a combined Rayleigh fractionation and binary mixing model and utilising a pristine mantle-type starting composition, the elevated melt δ^18^O values at Merapi and Kelut can be replicated by invoking ca. 10 % fractional crystallisation accompanied by a maximum of 20 wt.% crustal assimilation. This result is similar to mixing models in δ^18^O–^87^Sr/^86^Sr space, where we can see that some of the clinopyroxene crystals at Batur and Kelut record less than 10 % mixing between a source contaminated parent melt and crustal lithologies, whereas some clinopyroxene grains at Merapi record slightly higher degrees of mixing, again with a maximum of 20 % (Fig. 3c). This amount of crustal assimilation would be manifest as detectable changes to the isotopic composition of rock, mineral, and fumarole emissions, as exemplified at Merapi^31,53,55,57,58^.

We also note here that a small number of clinopyroxene grains from Batur record both relatively high and low δ^18^O values (Fig. 3). Minor degrees of crustal assimilation involving both high δ^18^O material (e.g. volcanoclastic sediments) and low δ^18^O material (e.g. hydrothermally altered lower arc crust, see refs. ^34,35,52^) cannot therefore be excluded for Bali sector volcanoes, but this does not seem to be a major process at Agung, which shows a relatively tight range of clinopyroxene δ^18^O values.

### Arc-wide δ^18^O record of mafic minerals

The previously published oxygen isotope data for pyroxene from the Sunda arc were obtained by either conventional fluorination (CF) of powdered mineral separates^13,26,31^ or laser fluorination (LF) of single or several crystals^13,14,30,31,36,37^. The SIMS method differs in that it analyses a sample volume many orders of magnitude smaller than these methods and offers a high degree of spatial control during analysis. Here we compare our new SIMS data to the available LF data for the Sunda arc, disregarding data obtained on relatively large volume, pulverised bulk mineral separates analysed by CF in order to facilitate as direct a comparison as possible^12^ (cf. Fig. 1).

**Fig. 4:**
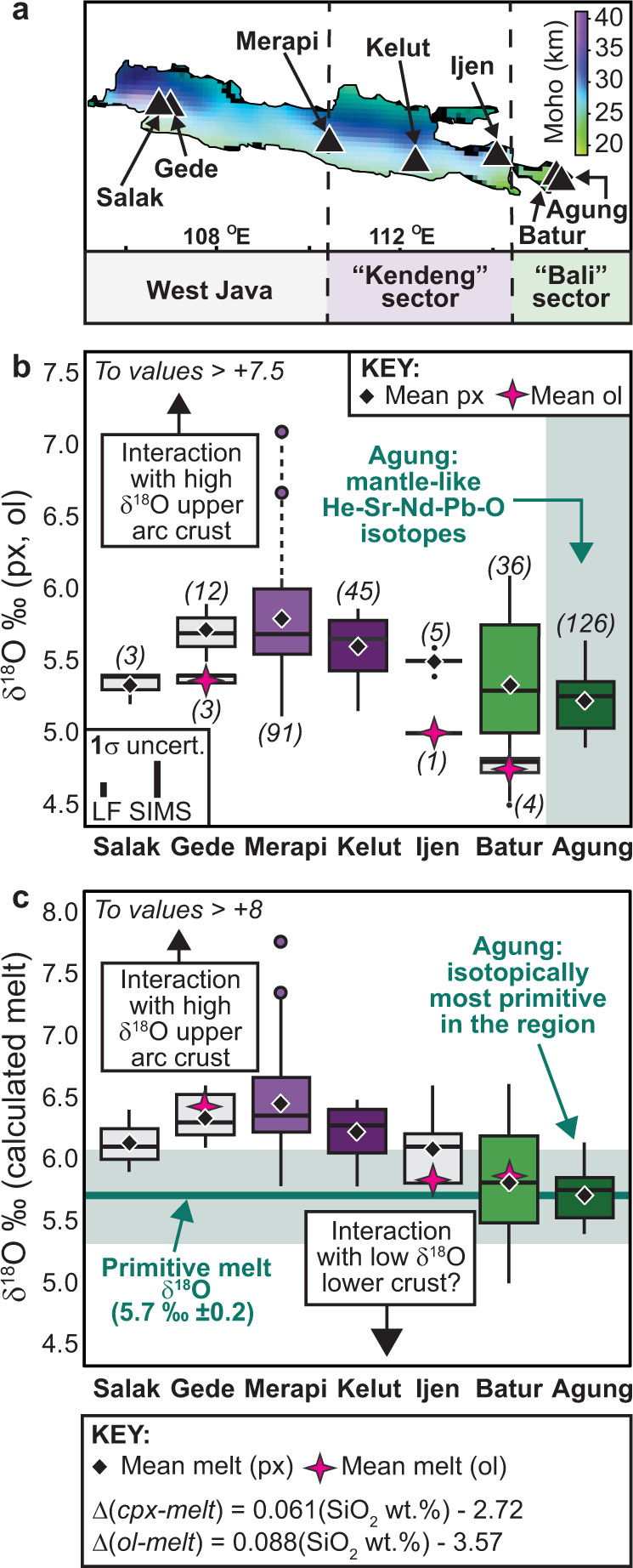
Arc-wide compilation of δ18O values in mafic minerals and equilibrium melts. **a** Map of Java and Bali showing Moho depths estimated from Bouguer gravity anomalies (after ref. ^40^). The Moho lies at ca. 18-20 km beneath Bali compared to ca. 25-30 km beneath Central to East Java. **b** Box-and-whisker plots of pyroxene and olivine δ^18^O values from volcanoes along the Java-Bali segment of the Sunda arc, showing grain averaged SIMS clinopyroxene data for Merapi, Kelut, Batur, and Agung (dark grey), pyroxene LF data for Salak^36^, Gede^30^, and Ijen^37^, and olivine LF data for Gede^30^, Ijen^37^, and Batur (ref. ^14^ and this study). The number of analyses per volcano are given in parentheses. **c** Box-and-whisker plots of equilibrium melt δ^18^O values calculated for the mineral data shown in panel **b** by employing the silica-dependent fractionation formulations of ref. ^24^. The clinopyroxene data suggest a parental melt δ^18^O value of 5.7 ‰ (±0.2 1sd) for the Sunda arc. For most Javanese volcanoes, magmas are stored in the arc crust where they evolve while assimilating crustal materials, resulting in variably elevated δ^18^O values in clinopyroxene. Data plotting was performed using the “ggplot2” package available via CRAN (Comprehensive R Archive Network; https://cran.r-project.org/). Abbreviations: cpx, clinopyroxene; ol, olivine; px, pyroxene.

Looking at East Java, the literature data for Ijen volcano^37^ fit into the step-wise decrease in mean pyroxene δ^18^O values from west to east, giving a sequence of: Merapi = 5.8 ‰, Kelut = 5.6 ‰, Ijen = 5.5 ‰, Batur = 5.3 ‰, and Agung = 5.2 ‰ (Fig. 4). This is consistent with the idea that pyroxene formed principally within the deeper parts of the magma plumbing system at Ijen, with minimal interaction with the arc crust reflected in pyroxene δ^18^O values^37,52^. In West Java, pyroxene grains from Gede volcano yielded a mean δ^18^O value of 5.7 ‰ (ref. ^30^), similar to Merapi. Two-pyroxene geothermobarometry has shown that Gede pyroxene crystallised at 13–24 km depth within the arc crust^30^, which is similar to pyroxene crystallisation depths of 9–21 km depth calculated for the Merapi 2006 eruption^12^, thus offering a possible explanation for their similar δ^18^O values. Pyroxene grains from Salak volcano, on the other hand, record a relatively low mean δ^18^O value of 5.3 ‰ (ref. ^36^), more akin to the δ^18^O values observed on Bali. We cannot fully assess whether Salak pyroxenes truly record mantle-like δ^18^O values, as only three analyses are available. However, it is possible that either i) a relatively unmodified δ^18^O value was sampled at Salak^36^ or ii) Salak magma assimilated ^18^O-depleted lower arc crust whose δ^18^O values were modified by high temperature alteration^52^. Assimilation of low δ^18^O hydrothermally altered crust has been suggested based on a small number of low δ^18^O values in plagioclase at Merapi^35^, however, in the case of Merapi, the dominant crustal influence appears to come from ^18^O-enriched crustal sedimentary rocks^31^ (Fig. 3c, d). In summary, oxygen isotope heterogeneity is introduced via magma–crust interaction involving various types of crustal materials to an increasing degree as we move westwards from Agung to Central Java (Fig. 4). This crustal influence is greatest in Central Java where clinopyroxene δ^18^O values peak at 7.1 ‰ at Merapi and decreases towards West Java. The Central Java δ^18^O “high” appears to coincide geologically with the Kendeng sedimentary basin in Central and East Java, which contains >6 km low density volcaniclastic and sedimentary rocks^59^ (Fig. 1). Mafic magma plumbing systems intersecting this sedimentary basin would likely foster magma–crust interaction causing relatively high δ^18^O values in clinopyroxene.

Due to a scarcity of olivine phenocrysts, there are very few olivine δ^18^O values from the Java-Bali segment of the Sunda arc. The published data include three values for Gede in West Java (mean of 5.4 ‰)^30^, one value for Ijen in East Java (5.0 ‰)^37^, and four values for Batur on Bali (mean of 4.7 ‰; ref. ^14^ and this study). Although the arc-wide olivine dataset is comparatively small, a decrease in mean δ^18^O olivine values from West Java to Bali can be observed, paralleling the trend in the pyroxene data (Fig. 4b). Melt δ^18^O values calculated from olivine at Ijen and Batur equal ca. 5.8 ‰ on average, which overlaps the composition of melts calculated from their respective clinopyroxene values (Fig. 4c).

A first-order conclusion of this study is that the δ^18^O values of clinopyroxene from volcanoes in the Central to East Sunda arc reflect variable degrees of enrichment in ^18^O from crustal oxygen, due to the preponderance of trans-crustal magma storage and subsequent clinopyroxene crystallisation in Java which promotes magmatic assimilation of arc crust. In contrast, clinopyroxene crystallisation occurs predominantly at the crust-mantle boundary beneath Bali (East Sunda arc), offering a unique window into both lower crustal assimilation (Batur) and primitive arc magma compositions (Agung). The clinopyroxene data from Agung are a particularly robust reflection of mantle-derived melts supplying the arc, because their δ^18^O values are correlated with independent isotopic markers of mantle derivation, including relatively low ^87^Sr/^86^Sr, ^206^Pb/^204^Pb, ^207^Pb/^204^Pb, ^208^Pb/^204^Pb, and high ^3^He/^4^He ratios. The results of this study lead us to conclude that Sunda arc primitive melts are not significantly metasomatised or enriched in ^18^O from the subducting slab and they possess a mean δ^18^O value of 5.7 ‰ (±0.2 1sd), in agreement with the δ^18^O range for MORB-type upper mantle-derived melts. The intersection of trans-crustal mafic plumbing systems and a relatively thick arc crust in parts of the Sunda arc (e.g. Central and East Java) exerts a major control in terms of modifying primary clinopyroxene δ^18^O values and causing elevated oxygen isotope ratios. Intracrystal oxygen isotope analysis of clinopyroxene thus offers an effective tool that, when applied systematically on a regional scale, can disentangle mantle from crustal oxygen compositions in arc systems.

## Methods

### Sample preparation

Crystal aliquots of clinopyroxene and olivine were separated from crushed lava samples under a binocular microscope and cleaned in pure ethanol in preparation for oxygen isotope analysis by laser fluorination (LF) and Secondary Ion Mass Spectrometry (SIMS). One split of crystals was analysed by LF (see below) and, in the case of clinopyroxene, another split was mounted on double-sided tape under an optical microscope, adjacent to a fragment of the reference material NRM-AG-1. The mounts were then cast in epoxy resin and polished using an automated polishing machine employing progressively fining diamond suspensions (down to 1 µm for the final polishing step). Care was taken to ensure that crystals were placed within a distance of >5 mm from the edge of the mount, and that the mount was polished flat with minimal relief in order to avoid analytical artefacts associated with sample geometry and topography^60,61^. Finally, the sample mounts were coated with carbon for Electron Probe Micro Analysis (EPMA), after which the carbon coat was removed from the sample mounts by gently polishing the surface with a 1 µm diamond solution. The sample mounts were then cleaned with pure ethanol and coated with a 20 nm gold layer prior to SIMS analysis. All sample mounts were imaged by Scanning Electron Microscopy (SEM) before analysis to create sample maps to aid the placement of SIMS analysis spots on inclusion-free crystal domains. The sample mounts were also inspected after SIMS analysis using optical microscopy and SEM to verify that the analysis pits did not intersect foreign material.

### Electron probe micro analysis (EPMA)

Mineral chemical data were acquired using the field-emission gun source JEOL JXA-8530F Hyperprobe (FEG-EPMA) at Uppsala University, Sweden following the protocols employed by ref. ^14^. The run conditions were 15 kV accelerating voltage and 10 nA probe current with 10 s on peak and 5 s on lower and upper background, with a beam diameter of 2 μm for pyroxene analysis. The following standards were used for calibration: wollastonite for Ca and Si, pyrophanite (MnTiO_3_) for Mn and Ti, magnesium oxide for Mg, orthoclase for K, albite for Na, aluminium oxide for Al, fayalite for Fe, nickel oxide for Ni, and chromium oxide for Cr. Analytical precision was measured on Smithsonian Institute mineral standards, including USNM 111312 (olivine), USNM 122142 (Cr-augite), USNM 137041 (anorthite), USNM 115900 (Ca-plagioclase), and USNM 133868 (anorthoclase). Uncertainties on the standards are as follows: SiO_2_, Al_2_O_3_, MgO and CaO ≤ 1.5% s.d., FeO ≤ 2.2% s.d., Na_2_O in plagioclase and clinopyroxene ≤ 4.5% s.d., and minor elements ≤ 10% s.d.

### Laser fluorination of minerals

Laser fluorination of mineral grains was carried out in the Department of Geological Sciences, University of Cape Town (UCT), South Africa, using mineral fragments weighing ca. 2 mg for each independent run, with the number of grains in each analysis varying from one to three. The results are reported in standard δ-notation relative to V-SMOW (Vienna Standard Mean Ocean Water), where δ = [(^18^O/^16^O)sample/(^18^O/^16^O)V-SMOW-1]*1000. Full analytical details of the laser fluorination method employed at UCT are given in ref. ^62^. Measured values of the UCT internal standard MON GT (Monastery garnet, δ^18^O = 5.38 ‰^62^) were used to normalise the raw data and correct for drift in the reference gas. The δ^18^O value of MON GT was established by cross-calibration with the UWG-2 garnet standard of ref. ^63^ and San Carlos olivine. The long-term average difference in δ^18^O values of duplicates of MON GT is 0.12 ‰ (*n* = 341), which corresponds to a 2σ value of 0.16 ‰.

### Secondary ion mass spectrometry (SIMS) analysis of clinopyroxene

Polished clinopyroxene crystals were analysed for their oxygen isotope ratios by SIMS at the NordSIMS ionprobe facility, Swedish Museum of Natural History, Stockholm, Sweden, using a CAMECA IMS 1280 multicollector equipped instrument. Most of the Batur and Agung clinopyroxene were analysed in January 2017, but an additional set of Agung clinopyroxene were analysed in December 2020. Kelut clinopyroxene were analysed in October 2017. The Merapi data were acquired in 2014 and reported in ref. ^12^ (and also tabulated in Supplementary Table 5 for completeness).

The SIMS instrumentation and methods employed in this study are based on refs. ^61,64^ and summarised here. A Cs+ primary beam of 1.5 nA was used in critically-focused mode together with a 5 or 10 μm raster to sputter a ca. 10 μm sample area with an impact energy of 20 keV. A normal incidence low energy electron gun provided charge compensation. The runs comprised a 60 second pre-sputter period with a raster of 20 μm, and secondary ion beam centreing in the field aperture using the ^16^O signal followed by 48 s (12 cycles of 4 s integrations) of data acquisition using two Faraday detectors in the multicollector system operating at a common mass resolution of ca. 2500. The secondary magnet field was regulated at high precision using a Metrolab NMR teslameter. To monitor external reproducibility during the analytical sessions, a sample-bracketing procedure was employed, whereby every block of four or five sample analyses was bracketed before and after by two analyses of augitic reference material NRM-AG-1 (with a δ^18^O value of 5.45, as determined by repeated LF analysis)^12^. In all analytical sessions, the data were corrected for within-session ratio drift and instrumental mass fractionation (IMF) using NRM-AG-1, as described in detail in ref. ^12^. All oxygen isotope ratios are reported in standard δ-notation.

Throughout the Batur and Agung 2017 analytical session, external precision was 0.27‰ (1 sd) on NRM-AG-1 and internal precision was on average 0.12 ‰ (1 se; overall range of 0.07 to 0.23 ‰). The average ^16^O intensity was 1.8 ×10^9^ cps. Throughout the Kelut analytical session, external precision was 0.14 ‰ (1 sd) on NRM-AG-1 and internal precision was on average 0.08 ‰ (1 se; overall range of 0.06 to 0.12 ‰). The average ^16^O intensity during the Kelut session was 2.8 ×10^9^ cps. For the Agung 2020 analytical session, external precision was 0.10 ‰ (1 sd) on NRM-AG-1 and internal precision was on average ±0.07 ‰ (1 se; overall range 0.06 to 0.11 ‰). The average ^16^O intensity was 2.7 ×10^9^ cps. Note that the 2020 analytical session utilised the recently installed low noise high-gain (10^12^Ω) Faraday amplifier at NordSIMS, which improves the signal/noise level for ^18^O. Reproducibility and precision during the Merapi analytical session is reported in ref. ^12^.

All mounts were analysed by optical microscopy after SIMS analysis to verify that analysis spots were not placed on fractures or foreign phases. No rejections were made on this basis, however, a total of 8 spot analyses were filtered from the Batur dataset due to their relatively high DTFA (field aperture) values, which were in the range -60 to -80. These high DTFA values might indicate a degree of beam misalignment in which isotope fractionation can occur at the entrance slit crossover^65^ and those spots were filtered purely as a precaution, even though we did not observe strong evidence for their isotope ratios being biased.

In the main text, we display and discuss mineral grain averaged SIMS datasets. Note that averaging the δ^18^O value of each grain before plotting reduced over-representation of outlying individual spot values and that the overall mean δ^18^O values per volcano remain almost unchanged, while the interquartile ranges per group change very little. In cases where only one analysis point was available for an individual grain, this value was assigned to the mineral grain in question for use in the mineral grain averaged plots. A detailed explanation of our data handling and a comparison between SIMS and LF datasets is provided in Supplementary Figure 2. We also emphasise that we observed no statistically significant relationship between clinopyroxene mean δ^18^O values and clinopyroxene mean major element composition (expressed as Wo, En, and Fs components; Supplementary Figure 4). Instrumental mass fractionation (IMF) during SIMS δ^18^O analysis of augitic clinopyroxene due to matrix effects associated with small variations in mineral Ca, Mg, or Fe contents thus appears to be negligible (cf. ref. ^66^).

### Sr–Nd–Pb isotopes in bulk lavas (University of Cape Town, South Africa)

Most of the radiogenic isotope data reported here were obtained in 2020 by Multicollector Inductively Coupled Plasma Mass Spectrometry (MC-ICPMS) at the Department of Geological Sciences, University of Cape Town (UCT), South Africa, including Sr–Nd–Pb isotope ratios for two samples of the Agung 1963 lava, one sample of the Batur 1974 lava, one sample of the Kelut 2007 dome-forming lava, and two samples of the Merapi 2006 lava. All analysed materials consisted of pulverised aliquots of fresh, bulk lava samples that were split from the same lava samples utilised for SIMS analysis of clinopyroxene phenocrysts. The sample preparation and analytical methods employed have previously been described in detail (e.g. ref. ^67^) and are summarised here. After sample dissolution, a Thermo iCap quadrupole inductively coupled plasma-mass spectrometer (ICP-MS) was employed for trace element analysis, following procedures similar to those described in ref. ^68^, with routine internal two-sigma analytical errors for individual analyses < 3% RSD. For isotope ratios, elemental separation chemistry followed routines after ref. ^69^. All isotope ratio analyses were conducted using a Nu Instruments NuPlasma, with the addition of a DSN-1000 desolvating nebuliser during Nd and Pb analyses. ^87^Sr/^86^Sr data were referenced to a value of 0.710255 for SRM 987, with all ratios corrected for interference on mass 87 using the measured ^85^Rb signal and the natural ^87^Rb/^85^Rb ratio. Instrumental mass fractionation was corrected for using the exponential law and a value of 0.1194 for ^86^Sr/^88^Sr. ^143^Nd/^144^Nd data were referenced to a value of 0.512155 for JNdi-1, with all ratios corrected for Sm and Ce interferences using measured ^147^Sm and ^140^Ce signals and the natural isotope ratios. Instrumental mass fractionation was corrected for using the exponential law and a value of 0.7219 for ^146^Nd/^144^Nd. All Pb isotope data were referenced to values for SRM 981 of ^208^Pb/^204^Pb, ^207^Pb/^204^Pb, ^206^Pb/^204^Pb using normalising values of 36.7219, 15.4963, 16.9405. Instrumental mass fractionation was corrected for using the exponential law and a ^205^Tl/^203^Tl value of 2.3889 for SRM 997 added to all standards and samples (Pb:Tl of ±10:1). The andesitic reference material GSJ-JA-2 was analysed as an unknown alongside the samples in this study and gave the following ratios: ^87^Sr/^86^Sr = 0.706406 ± 0.000013; ^143^Nd/^144^Nd = 0.512543 ± 0.000012; ^208^Pb/^204^Pb = 38.6703 ± 0.0029, ^207^Pb/^204^Pb = 15.6054 ± 0.0010, ^206^Pb/^204^Pb = 18.4008 ± 0.0009. These results are in good agreement with accepted values from the GeoReM database (http://georem.mpch-mainz.gwdg.de; ref. ^70^). Total procedural blanks in the radiogenic isotope facility at the University of Cape Town are < 250 pg Sr; < 100 pg Nd and < 200 pg Pb and are insignificant relative to the concentrations in Indonesian lavas. Thus, no blank corrections were applied.

### Sr–Nd isotopes in bulk lavas (Vrije Universiteit Amsterdam, Netherlands)

In addition to the samples analysed at UCT, one sample of each of the Kelut 2007 dome-forming lava and the Merapi 2006 lava were analysed in 2011 for trace element concentrations by ICP-MS, Sr isotope ratios by Thermal Ionisation Mass Spectrometry (TIMS), and Nd isotope ratios by MC-ICPMS at the Vrije Universiteit Amsterdam (VUA), Netherlands. These data formed part of the MSc thesis of M. van Helden^71^. The methods employed are briefly described here. After sample dissolution, trace element analysis was carried out on a Thermo X-Series II Quadrupole ICP-MS. Instrumental drift and reproducibility were monitored using repeated measurements of the BHVO-2 and BCR-2 geological reference materials. Analytical precision was better than 10% for all elements. Strontium separation was performed using conventional Sr Spec cation exchange chromatography^69^ and isotope measurements were subsequently performed on a Finnigan MAT 262 TIMS system operating in static mode. Instrumental mass fractionation was corrected by normalising to ^86^Sr/^88^Sr = 0.1194 using an exponential correction law. Measurements of NBS 987 made during the course of this study were within error of the long-term reproducibility at VUA. Two BHVO-2 standards were processed with the samples and gave an average ^87^Sr/^86^Sr ratio of 0.703472 ± 0.000009 (2SE). Procedural blank values at the VU are typically ≤ 100 pg, which is negligible when compared to the 500 ng Sr loaded per sample and therefore no blank corrections were made. Neodymium separation was carried out as a two-column procedure, utilising conventional TRU Spec and LN Spec cation exchange chromatography^69^. Neodymium isotopes were analysed using a Finnigan Neptune MC-ICPMS, employing the method in ref. ^72^. During the course of this study, repeat analysis of the CIGO internal standard gave a mean ^143^Nd/^144^Nd ratio of 0.511349 ± 0.000010 (2SE, *n* = 13) in agreement with previous TIMS measurements of the CIGO standard of 0.511342 ± 0.000007 (2SE, *n* = 18)^73^. Repeated analyses (n = 2) of BHVO-2 gave a mean ^143^Nd/^144^Nd ratio of 0.512962 ± 0.000007 (2SE). Nd blanks were less than 10 ppb which is negligible compared to the 200–400 ppb (Nd) measuring solutions used and hence no blank corrections were applied.

## Supplementary information

Supplementary Information

## Data Availability

The authors declare that all relevant data, including source data, are available within the article and its supplementary information files.
