# Supplementary material for: Sunda arc mantle source δ18O value revealed by intracrystal isotope analysis
Source: Nat Commun. 2021 Jun 24;12:3930. doi: 10.1038/s41467-021-24143-3 (PMC8225799; doi:10.1038/s41467-021-24143-3)
Supplement: Supplementary file 1 — Supplementary Information [file 41467_2021_24143_MOESM1_ESM.pdf]

Sunda arc mantle source $\delta^{18}O$ value revealed by intracrystal isotope analysis

Frances M. Deegan, Martin J. Whitehouse, Valentin R. Troll, Harri Geiger, Heejin Jeon, Petrus leRoux, Chris Harris, Marcel van Helden, Osvaldo González-Maurel

Supplementary Information

__Contents__:

Supplementary Figure 1: Clinopyroxene mineral chemistry.

Supplementary Figure 2. Comparison between SIMS and LF data.

Supplementary Figure 3. Sr-Nd-Pb isotope diagrams.

Supplementary Figure 4: Oxygen isotopes versus clinopyroxene components.

Supplementary Discussion: Section 1: Comparison between SIMS and LF data. Section 2: Accuracy and internal consistency of the SIMS data.

Supplementary Table 1: Summary of oxygen isotope data for clinopyroxene obtained by SIMS.

Supplementary Table 2: Sr-Nd-Pb isotopic data for the bulk lava sample splits corresponding to the SIMS samples.

Supplementary Table 3: Summary of oxygen isotope data for mafic minerals obtained by LF and their calculated equilibrium melt values.

Supplementary Table 4: Source data 1 (EPMA).

Supplementary Table 5: Source data 2 (SIMS).

Supplementary References.

Correspondence/request for materials to: frances.deegan@geo.uu.se

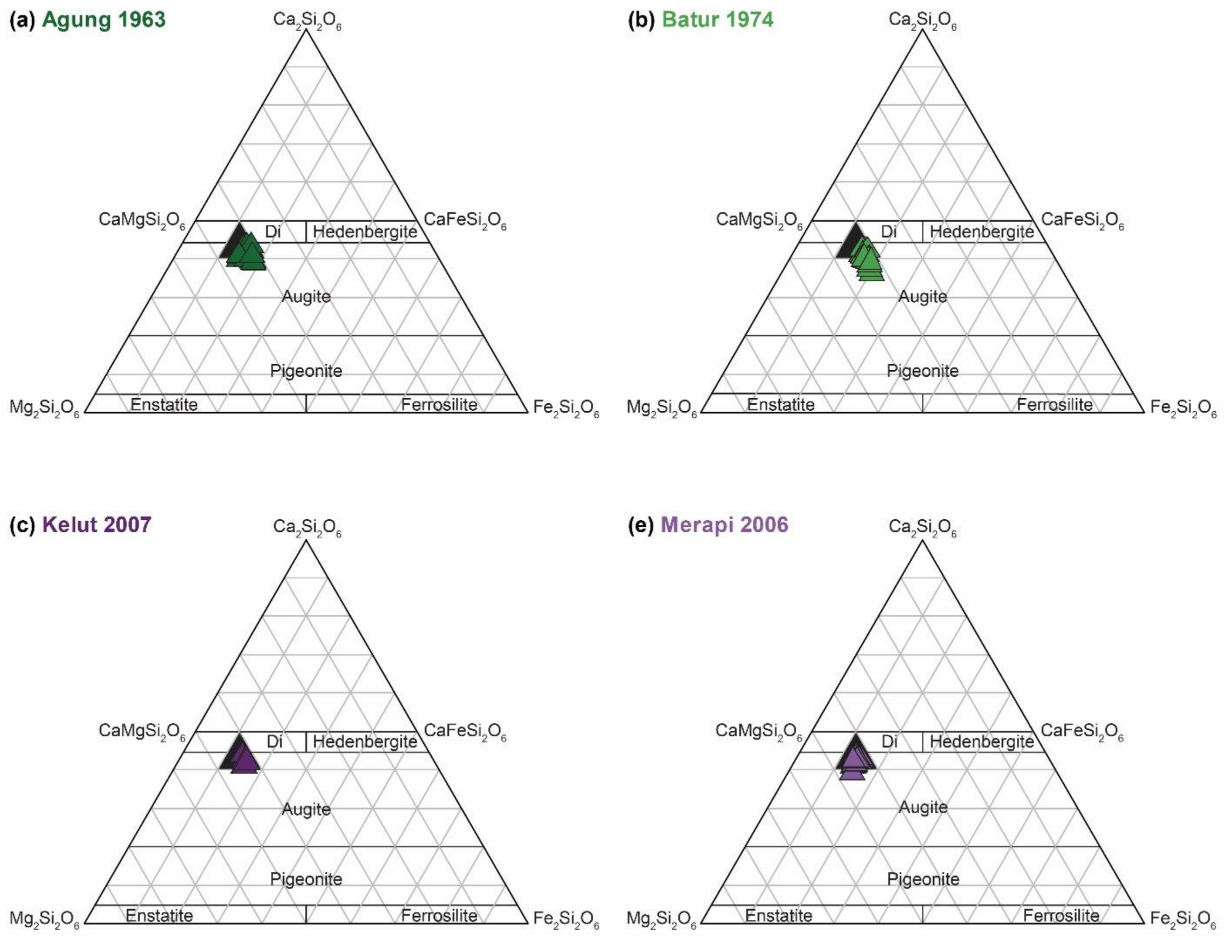

Supplementary Figure 1. Clinopyroxene mineral chemistry. Composition of clinopyroxene grains analysed by SIMS in this study from **(a)** Agung, **(b)** Batur, **(c)** Kelut, and **(d)** Merapi as determined by EPMA (see **Table S4** for full dataset). The black shaded area represents the compositional field of SIMS augitic reference material NRM-AG-1 after ref.[1].

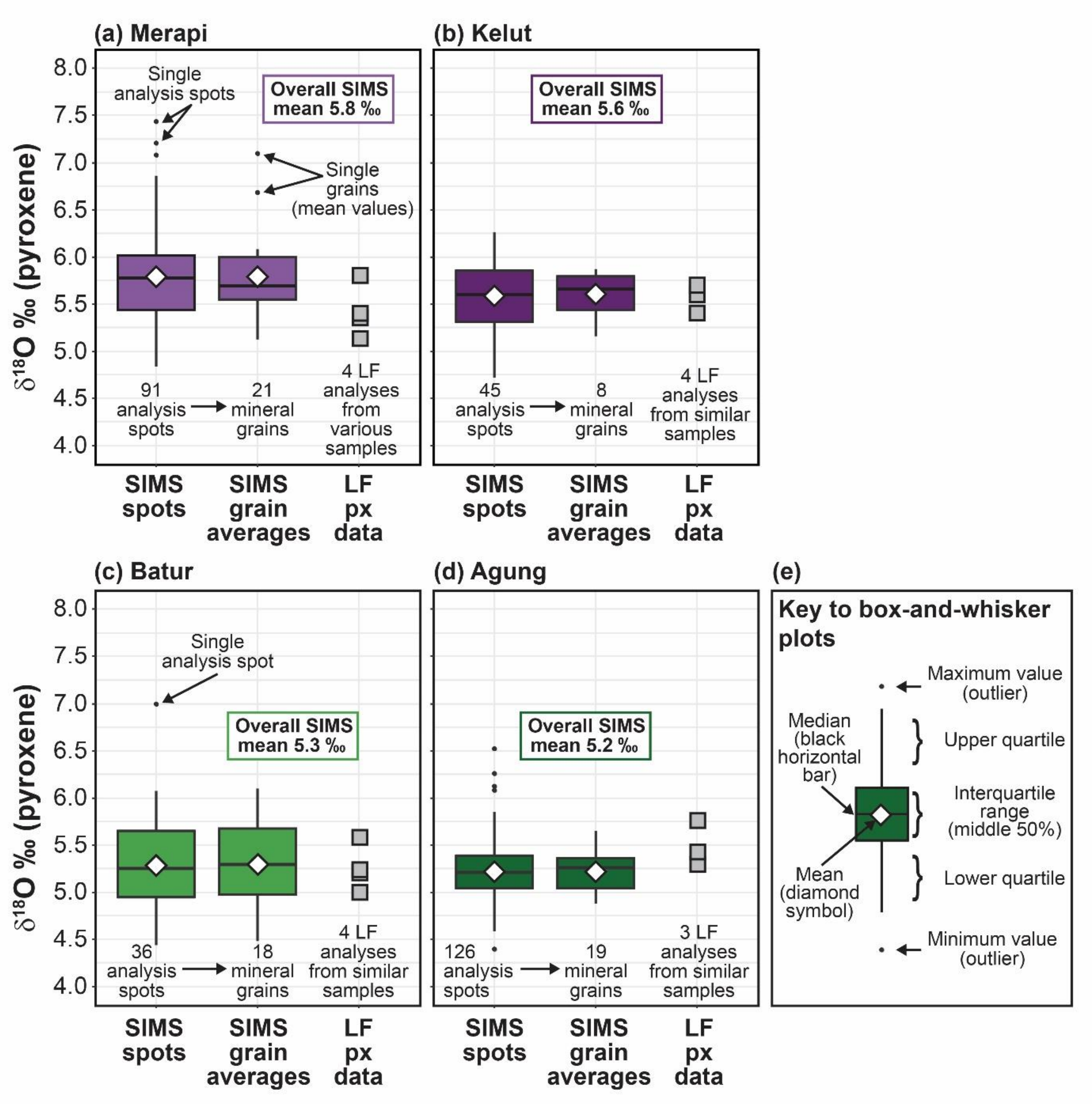

Supplementary Figure 2. Comparison between SIMS and LF data. SIMS data from **(a)** Merapi, **(b)** Kelut, **(c)** Batur, and **(d)** Agung are shown as box-and-whisker plots calculated using individual SIMS analyses (left) and using the mean $\delta^{18}O$ values of each mineral grain (middle part of each panel). In cases where only one analysis point was available for an individual grain, this value was assigned to the mineral grain in question for use in the mineral grain averaged plots. The LF data are shown to the right of each panel and are plotted as individual data points due to the small amount of available analyses. In the main text file, we display and discuss the mineral grain averaged SIMS datasets. Note that averaging the $\delta^{18}O$ value of each grain before plotting reduced over-representation of outlying values and that the overall mean $\delta^{18}O$ values per group (white diamond symbols) remain almost unchanged, while the interquartile ranges per group change very little. A guide to reading the presented SIMS data is provided in **(e)**. SIMS data calculations were performed using the “ggplot2” package

available via CRAN (Comprehensive R Archive Network; https://cran.r-project.org/). Average propagated uncertainties are 0.25‰ (1σ) for the SIMS data and 0.1‰ (1σ) for the LF data. Abbreviation: px, pyroxene.

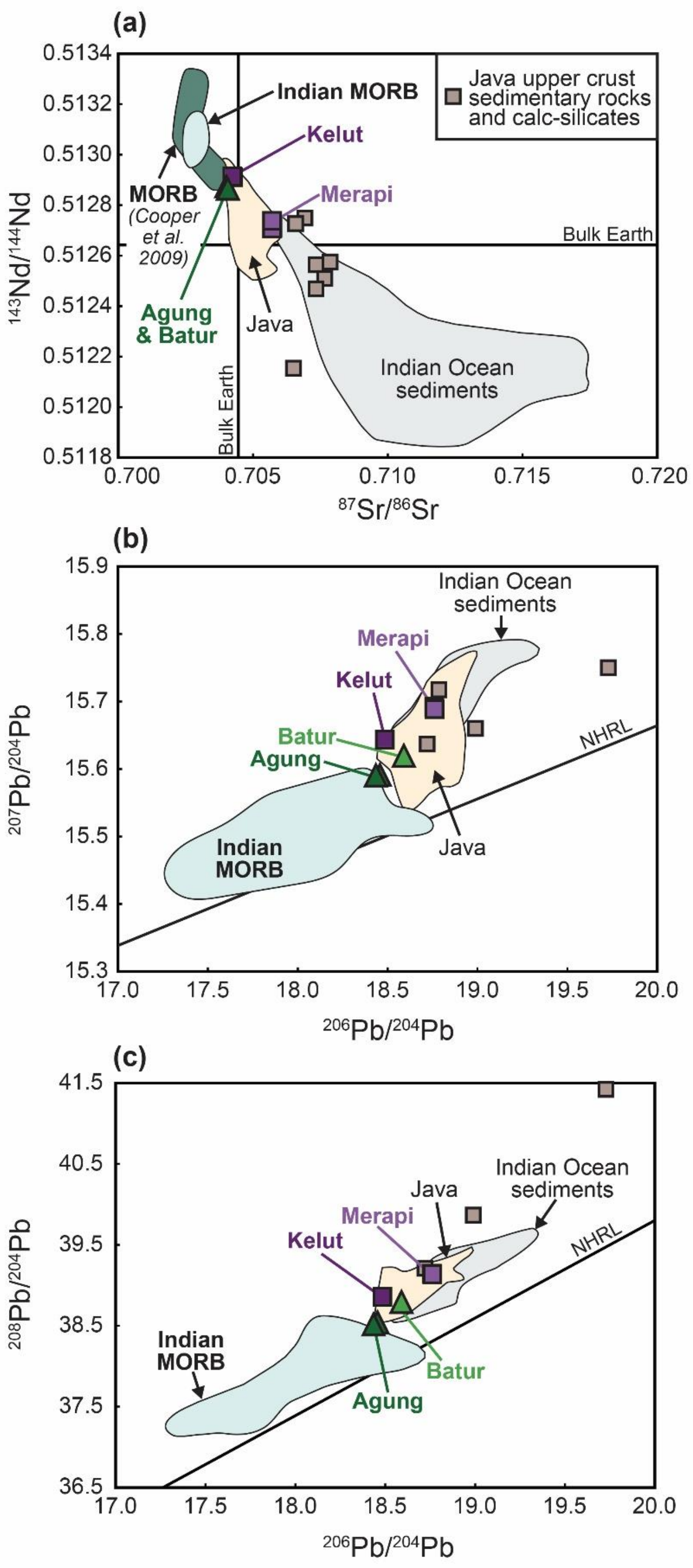

Supplementary Figure 3. Sr-Nd-Pb isotope diagrams. (a) $^{143}Nd/^{144}Nd$ versus $^{87}Sr/^{86}Sr$, **(b)** $^{207}Pb/^{206}Pb$ versus $^{206}Pb/^{204}Pb$, and **(c)** $^{208}Pb/^{204}Pb$ versus $^{206}Pb/^{204}Pb$ isotope diagrams showing

data obtained from lava samples utilised in this study (**Table S2**) compared to other regional data. Data fields for Indian MORB, Java, and Indian Ocean sediments are redrawn after ref.[2] and references therein. Additional data for MORB are from ref.[3]. Data for upper arc crust samples are from refs.[2,4]. The Northern Hemisphere Reference Line (NHRL) is drawn after ref.[5]. Uncertainties on the isotopic data presented in this study are smaller than the symbol sizes. These diagrams illustrate that lavas from the 1963 eruption of Agung are among the most isotopically primitive erupted materials in the Java-Bali segment of the Sunda arc.

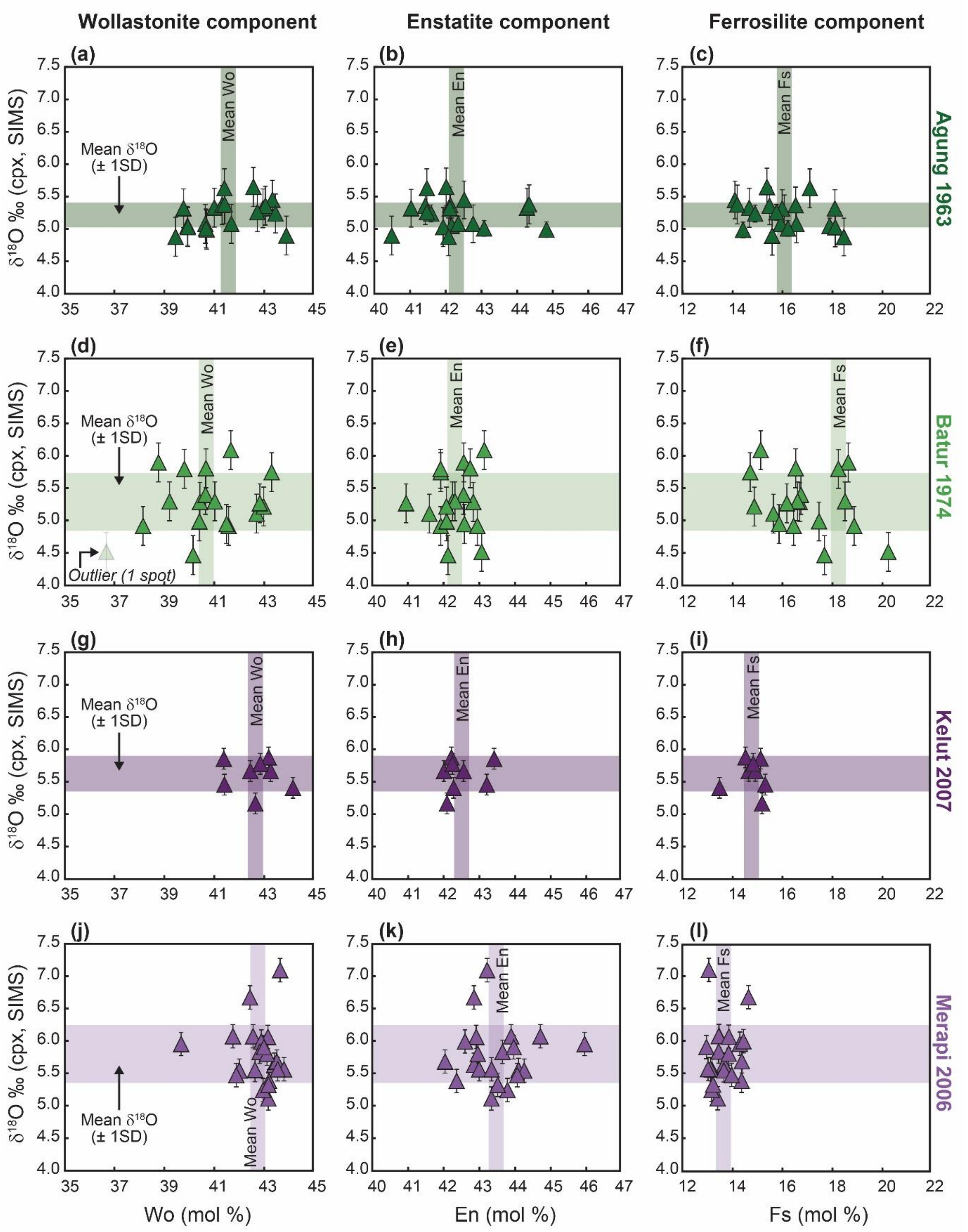

Supplementary Figure 4. Oxygen isotopes versus clinopyroxene components. Grain-averaged clinopyroxene $\delta^{18}O$ values obtained by SIMS versus Wo, En, and Fs contents of the corresponding grains from Agung (**a** to **c**), Batur (**d** to **f**), Kelut (**g** to **i**), and Merapi (**j** to **l**).

There is no statistically significant relationship between clinopyroxene mean $\delta^{18}O$ values and clinopyroxene mean major element composition (expressed as Wo, En, and Fs components). Instrumental mass fractionation (IMF) during SIMS $\delta^{18}O$ analysis of augitic clinopyroxene due to matrix effects associated with small variations in mineral Ca, Mg, or Fe contents thus appears to be negligible (cf. ref.[6]). Note that we have identified an outlier grain in the Batur dataset, which represents just one single SIMS analysis point and cannot be employed to draw conclusions about IMF. Error bars represent 1σ uncertainties on the SIMS data. Abbreviations: cpx, clinopyroxene; En, enstatite; Fs, ferrosilite; Wo, wollastonite

Supplementary Discussion

Section 1: Comparison between SIMS and LF data

Here we compare oxygen isotope data obtained for clinopyroxene grains by SIMS and by LF for each of the individual volcanoes studied. The SIMS oxygen isotope datasets discussed below consist of the mean $\delta^{18}O$ values obtained per individual clinopyroxene grain, as reported in **Table S1 and Fig. S2**. The full SIMS dataset (all individual analyses) is reported in **Table S5**.

- **Merapi.** SIMS analysis of clinopyroxene yielded mean $\delta^{18}O$ values per mineral grain ranging from 5.1 to 7.1 ‰ (mean of 5.8 ‰, n = 91 spot analyses from 21 grains; see **Table S1**). There are currently four published LF values for pyroxene from Merapi[7]. One of the pyroxene samples analysed by LF is from the 2006 lava (5.4 ‰; shown in **Table S3**), one is from the 2010 lava (5.1 ‰), and two are from a plutonic sample (5.4 and 5.8 ‰). The range of LF and SIMS $\delta^{18}O$ values overlap, but there is an absence of relatively high $\delta^{18}O$ values in the LF dataset. This may simply be due to the small number of minerals analysed by LF (just four samples), which happened to not include any high-$\delta^{18}O$ material. Alternatively, this may be due to low $\delta^{18}O$ magnetite inclusions in pyroxene, which are difficult to discern from host pyroxene as both minerals appear dark and opaque under the binocular microscope[1]. Magnetite inclusions are frequent in the Merapi pyroxene utilised in this study and it is thus conceivable that they are present in the 2010 lava and plutonic samples too.
- **Kelut.** SIMS analysis of clinopyroxene yielded $\delta^{18}O$ values ranging from 5.2 to 5.9 ‰ (mean of 5.6 ‰, n = 45 spot analyses from 8 crystals; see **Table S1**). The LF data for Kelut were obtained from similar samples to those analysed by SIMS and the results range from 5.4 to 5.7 ‰ (mean of 5.5 ‰, n = 4, ref.[8]; see **Table S3**). There is thus excellent agreement between the LF and SIMS data.
- **Batur.** SIMS analysis of Batur clinopyroxene yielded $\delta^{18}O$ values ranging from 4.5 to 6.1 ‰ (mean of 5.3 ‰, n = 36 spot analyses from 18 crystals; see **Table S1**). The LF data range from 5.0 to 5.6 ‰ (mean of 5.3 ‰, n = 4, ref.[9] and this study; see **Table S3**) for Batur 1963 and 1974 lavas. There is thus excellent agreement between the LF and SIMS data, with the SIMS data additionally recording a small number of relatively high and low outlying values.
- **Agung.** SIMS analysis of clinopyroxene yielded $\delta^{18}O$ values ranging from 4.9 to 5.7 ‰ (mean of 5.2 ‰, n = 126 spot analyses from 19 crystals; see **Table S1**). The LF data range from 5.3 to 5.8 ‰ (mean of 5.5 ‰, n = 3; ref.[9] and this study; see **Table S3**). The SIMS and LF data thus agree well, although the mean $\delta^{18}O$ value for Agung pyroxene obtained by LF is slightly high compared to the mean of the SIMS dataset. This is due to one relatively high $\delta^{18}O$ value of 5.8 ‰ obtained by LF for Agung, which, in a pool of just three LF data points, results in a relatively high LF mean $\delta^{18}O$ value.
- **Bali overall.** Note that when the Batur and Agung SIMS data are combined to form an overall "Bali sector" dataset, the mean $\delta^{18}O$ values obtained for pyroxene by SIMS versus those determined by LF are statistically indistinguishable, differing by only 0.1 ‰.

Section 2: Accuracy and internal consistency of the SIMS data

The available LF data plotted in **Fig. S2** are relatively few in number and were generated at various laboratories. Moreover, in the case of Merapi, the LF data were generated on volcanic and plutonic samples that are not always directly comparable to the Merapi lava sample utilised in our SIMS clinopyroxene study. These combined factors can explain why the LF data have so far failed to show a clear west to east trend of decreasing $\delta^{18}O$ values that is observed in our SIMS data (see main text **Figs. 3 and 4**). The SIMS data presented here were all collected at the same laboratory, utilising the same analytical protocol, and the same reference material. Our SIMS dataset is thus internally consistent and agrees well with external (LF) data, particularly where the LF data were obtained on similar samples to the SIMS samples. We therefore argue that our SIMS data are accurate and that they moreover allow us to capture an arc-wide trend in clinopyroxene $\delta^{18}O$ values that was previously not observable due to the lack of a single, arc-wide, systematic and internally consistent study. Furthermore, such a study was not possible until now due to a paucity of suitable reference materials for SIMS clinopyroxene $\delta^{18}O$ analysis, which was recently remedied by the author team[1].

Table S1 Summary of grain averaged oxygen isotope data for clinopyroxene obtained by SIMS.

Mount/grain	$\delta^{18}O$ ‰ clinopyroxene*	$\delta^{18}O$ ‰ melt†	Mount/grain	$\delta^{18}O$ ‰ clinopyroxene*	$\delta^{18}O$ ‰ melt†
Agung 1963 lava (sample A-63-1)			***Batur 1974 lava (sample B-74-1)***		
1557/28	5.0 (6)	5.5	1557/6	5.3 (4)	5.8
1557/29	5.1 (6)	5.5	1557/7	5.3 (4)	5.8
1557/30	5.3 (2)	5.8	1557/12	5.4 (8)	5.9
1557/31	5.3 (2)	5.8	1557/13	5.3 (1)	5.8
1557/32	5.5 (1)	5.9	1557/14	5.1 (2)	5.6
1557/33	5.7 (2)	6.1	1557/15	5.8 (1)	6.3
1557/34	5.4 (2)	5.9	1557/16	6.1 (1)	6.6
1557/35	5.4 (2)	5.8	1557/17	5.2 (2)	5.7
1557/36	5.0 (2)	5.5	1557/18	4.9 (1)	5.4
1557/37	5.1 (14)	5.6	1557/19	4.5 (1)	5.0
1557/38	5.2 (6)	5.7	1557/20	5.0 (2)	5.5
1557/39	5.1 (6)	5.6	1557/21	5.3 (1)	5.8
1557/40	5.3 (6)	5.7	1557/22	5.0 (2)	5.5
1557/41	5.0 (6)	5.5	1557/23	4.5 (1)	5.0
1557/42	4.9 (10)	5.4	1557/24	4.9 (2)	5.4
1557/43	4.9 (5)	5.4	1557/25	5.9 (1)	6.4
1557/44	5.3 (15)	5.8	1557/26	5.8 (1)	6.3
1557/45	5.4 (20)	5.9	1557/27	5.8 (1)	6.3
1557/46	5.6 (13)	6.1			
Kelut 2007 dome-forming lava (sample K-08-1)			***Merapi 2006 lava (sample M-06-1)***		
1121/4	5.9 (5)	6.5	1083/4	5.3 (2)	5.9
1121/6	5.8 (4)	6.4	1083/6	5.4 (11)	6.1
1121/17	5.9 (9)	6.5	1083/7	5.1 (3)	5.8
1121/18	5.7 (4)	6.3	1083/12	5.6 (10)	6.2
1121/28	5.2 (9)	5.8	1083/16	5.9 (2)	6.6
1121/29	5.4 (4)	6.0	1083/17	5.8 (2)	6.5
1121/30	5.4 (6)	6.0	1083/21	6.1 (11)	6.7
1121/31	5.7 (4)	6.3	1083/22	7.1 (2)	7.8
			1083/24	5.6 (4)	6.2
			1083/25	5.6 (3)	6.2
			1083/26	5.7 (5)	6.4
			1083/27	5.3 (4)	6.0
			1083/29	5.6 (1)	6.3
			1083/32	5.5 (3)	6.1
			1083/33	6.0 (1)	6.7
			1083/34	5.8 (2)	6.5
			1083/35	5.6 (3)	6.2
			1083/36	6.1 (2)	6.7
			1083/37	6.1 (14)	6.7
			1083/38	6.7 (3)	7.3
			1083/39	6.0 (3)	6.6

*Values shown are the mean for each crystal; figures in parentheses are the number of spot analyses per clinopyroxene grain. Raw SIMS data including propagated uncertainties on individual spot analyses are provided in **Table S5**. †Melt values calculated by employing the bulk lava SiO_2 content (**Table S2**) and the silica-dependent formulations of ref.[10] Merapi values are calculated from data in ref.[1] (tabulated in **Table S5**).

Table S2 Sr-Nd-Pb isotopic data for the bulk lava sample splits corresponding to the SIMS samples.

Lava sample	SiO_2 (wt.%)	$^{87/86}Sr$	Sr (µg/g)	$^{143/144}Nd$	Nd (µg/g)	$^{208/204}Pb$	$^{207/204}Pb$	$^{206/204}Pb$	Pb (µg/g)	Method for isotope analysis (laboratory)
Agung 1963 lava										
A-63-1	52.5*	0.704069 (0.000012)	444	0.512865 (0.000014)	14	38.5377 (0.0034)	15.5909 (0.0011)	18.4568 (0.0009)	5	MC-ICPMS (UCT)
A-63-2	52.5*	0.704065 (0.000015)	443	0.512866 (0.000010)	14	38.5140 (0.0032)	15.5904 (0.0010)	18.4342 (0.0011)	5	MC-ICPMS (UCT)
Batur 1974 lava										
B-74-1	53.0*	0.704013 (0.000009)	556	0.512875 (0.000012)	13	38.7921 (0.0026)	15.6196 (0.0006)	18.5897 (0.0008)	5	MC-ICPMS (UCT)
Kelut 2007 dome-forming lava										
K-08-1	54.6**	0.704254 (0.000013)	597	0.512911 (0.000020)	7	38.8563 (0.0027)	15.6435 (0.0009)	18.4839 (0.0010)	8	MC-ICPMS (UCT)
KELUT-1-w	54.6**	0.704234 (0.000007)	623	0.512915 (0.000007)	8	-	-	-	-	TIMS and MC-ICPMS (VUA)
Merapi 2006 lava										
M-06-1	55.5#	0.705727 (0.000011)	585	0.512712 (0.000012)	16	39.1406 (0.0030)	15.6921 (0.0010)	18.7586 (0.0010)	18	MC-ICPMS (UCT)
M-06-4	55.2#	0.705713 (0.000010)	761	0.512708 (0.000013)	17	39.1348 (0.0034)	15.6889 (0.0010)	18.7609 (0.0010)	20	MC-ICPMS (UCT)
Merapi whole rock 1	55.5#	0.705727 (0.000009)	556	0.512738 (0.000008)	19	-	-	-	-	TIMS and MC-ICPMS (VUA)

Values in parentheses are 2σ uncertainties. SiO_2 data are from refs. [9] (*), [8] (**), [7] (#). Abbreviations: MC-ICPMS, Multicollector Inductively Coupled Plasma Mass Spectrometry; TIMS, Thermal Ionisation Mass Spectrometry; UCT, University of Cape Town (South Africa); VUA, Vrije Universiteit Amsterdam (Netherlands).

Table S3 Summary of oxygen isotope data for mafic minerals obtained by LF and their calculated equilibrium melt values.

Sample and type	$\delta^{18}O$ ‰ mineral	$\delta^{18}O$ ‰ melt	SiO_2 wt.% (bulk lava)	Reference
Agung 1963 lava				
A-BA1-63 pyroxene	5.4	5.9	52.5	Geiger *et al*., 2018[9]
A-BA1-63 pyroxene	5.3	5.8	52.5	Geiger *et al*., 2018[9]
A-BA1-63 pyroxene	5.8	6.2	52.5	Geiger *et al*., 2018[9]
Batur 1963 lava				
B-BA1-63 pyroxene	5.2	5.7	53.1	Geiger *et al*., 2018[9]
B-BA1-63 pyroxene	5.2	5.8	53.1	This study ($\delta^{18}O$) and Geiger *et al.*, 2018[9] (SiO_2)
B-BA-1-63 olivine	4.8	5.9	53.1	Geiger *et al*., 2018[9]
B-BA-1-63 olivine	4.9	6.0	53.1	Geiger *et al*., 2018[9]
Batur 1974 lava				
B-BA2-74 pyroxene	5.0	5.5	53.0	Geiger *et al*., 2018[9]
B-BA2-74 pyroxene	5.6	6.1	53.0	Geiger *et al*., 2018[9]
B-BA2-74 olivine	4.5	5.6	53.0	This study ($\delta^{18}O$) and Geiger *et al.*, 2018[9] (SiO_2)
B-BA2-74 olivine	4.8	5.9	53.0	This study ($\delta^{18}O$) and Geiger *et al.*, 2018[9] (SiO_2)
Kelut 2007 dome-forming lava				
K-BA-08			54.6	Jeffery *et al*., 2013[8]
K-L-6	5.4	6.0	54.9	Jeffery *et al*., 2013[8]
K-L-6	5.7	6.3	54.9	Jeffery *et al*., 2013[8]
K-A-1	5.4	6.0	55.0	Jeffery *et al*., 2013[8]
K-A-1	5.6	6.2	55.0	Jeffery *et al*., 2013[8]
Merapi 2006 lava				
M07-53	5.4	6.0	55.4	Troll *et al*., 2013[7] ($\delta^{18}O$) and Preece, 2014[11] (SiO_2)

Melt $\delta^{18}O$ values were calculated by employing the bulk lava SiO_2 content that links to the mineral $\delta^{18}O$ vales and the silica-dependent formulations of ref.[10]. Typical 2σ analytical uncertainty on the oxygen isotope data is 0.2 ‰.

Table S4 Source data 1. Mineral chemistry obtained by EPMA for clinopyroxene from the studied lava samples.

Mount/grain	SiO_2	TiO_2	Al_2O_3	FeO	MnO	CaO	Na_2O	Cr_2O_3	V_2O_3	Total	Wo	En	Fs
					Weight %							End-members	
Agung 1963 clinopyroxene													
1557/28	52.07	0.41	2.18	8.97	0.33	19.77	0.24	0.02	0.09	99.75	40.71	44.87	14.42
1557/29	50.49	0.66	3.09	10.96	0.39	19.10	0.41	0.00	0.05	99.66	39.93	42.18	17.89
1557/30	50.13	0.86	3.66	9.79	0.34	20.55	0.35	0.00	0.07	99.91	42.98	41.03	15.98
1557/31	51.97	0.46	2.26	9.10	0.34	19.87	0.22	0.00	0.08	99.76	41.02	44.32	14.66
1557/32	50.19	0.67	3.52	8.68	0.17	20.85	0.26	0.06	0.08	99.20	43.37	42.54	14.09
1557/33	50.08	0.84	4.21	9.22	0.28	19.94	0.35	0.00	0.01	99.07	42.60	42.03	15.37
1557/34	50.73	0.74	3.50	10.01	0.34	19.54	0.39	0.03	0.13	99.78	41.33	42.14	16.53
1557/35	50.02	0.84	3.99	9.47	0.30	20.57	0.33	0.06	0.08	99.96	43.08	41.44	15.48
1557/36	50.64	0.59	3.09	11.13	0.38	19.16	0.40	0.02	0.07	99.94	39.94	41.94	18.11
1557/37	50.60	0.69	3.50	10.24	0.35	19.63	0.41	0.01	0.01	100.32	40.66	42.79	16.55
1557/38	50.13	0.71	4.15	9.13	0.24	20.80	0.34	0.03	0.10	100.00	43.49	41.60	14.90
1557/39	51.20	0.53	2.44	9.77	0.35	19.97	0.35	0.00	0.04	99.27	41.71	42.37	15.93
1557/40	50.97	0.74	3.81	9.73	0.26	20.61	0.32	0.00	0.11	100.92	42.76	41.48	15.76
1557/41	51.29	0.57	3.18	10.02	0.27	19.62	0.37	0.03	0.02	100.31	40.68	43.10	16.22
1557/42	50.09	0.73	3.60	9.53	0.25	20.97	0.26	0.02	0.05	99.39	43.93	40.49	15.58
1557/43	50.87	0.61	2.61	11.34	0.42	18.92	0.35	0.00	0.02	99.74	39.45	42.09	18.46
1557/44	51.30	0.56	2.26	11.14	0.42	19.11	0.36	0.00	0.00	99.73	39.77	42.13	18.10
1557/45	52.13	0.42	2.11	8.70	0.37	19.84	0.23	0.00	0.04	99.11	41.44	44.38	14.18
1557/46	50.42	0.67	3.47	10.43	0.28	19.73	0.42	0.00	0.09	99.70	41.43	41.48	17.09
Batur 1974 clinopyroxene													
1557/6	51.83	0.58	1.96	11.40	0.38	18.83	0.34	0.03	0.11	100.04	39.21	42.27	18.53
1557/7	51.48	0.54	2.09	10.33	0.37	19.51	0.28	0.00	0.07	99.59	40.44	42.85	16.71
1557/12	52.19	0.48	1.62	10.33	0.36	19.56	0.25	0.05	0.06	99.63	40.66	42.58	16.76
1557/13	51.15	0.53	2.27	10.34	0.25	19.92	0.28	0.02	0.07	99.62	41.04	42.34	16.63
1557/14	51.09	0.65	2.88	9.62	0.31	20.49	0.35	0.02	0.04	99.85	42.73	41.61	15.66
1557/15	51.24	0.66	2.72	9.12	0.23	20.95	0.29	0.04	0.03	99.90	43.34	41.94	14.73
1557/16	51.84	0.60	2.30	9.41	0.29	20.21	0.27	0.00	0.06	100.06	41.69	43.16	15.15
1557/17	51.47	0.61	2.60	9.24	0.24	20.83	0.25	0.00	0.03	99.92	43.01	42.09	14.89
1557/18	52.28	0.51	1.81	10.18	0.37	20.07	0.28	0.01	0.06	100.12	41.60	41.93	16.47
1557/19	51.74	0.64	1.89	12.44	0.37	17.56	0.23	0.00	0.09	99.80	36.65	43.09	20.26
1557/20	52.02	0.55	2.24	9.77	0.27	19.93	0.29	0.00	0.06	99.83	41.51	42.60	15.88

Mount/grain	SiO_2	TiO_2	Al_2O_3	FeO	MnO	CaO	Na_2O	Cr_2O_3	V_2O_3	Total	Wo	En	Fs
					Weight %							End-members	
1557/21	50.94	0.62	2.13	9.89	0.34	20.43	0.22	0.00	0.03	98.66	42.86	40.95	16.19
1557/22	51.35	0.62	2.16	10.81	0.33	19.50	0.25	0.03	0.04	99.71	40.41	42.10	17.49
1557/23	51.26	0.58	2.38	10.89	0.36	19.28	0.36	0.07	0.08	99.81	40.16	42.14	17.70
1557/24	51.46	0.51	1.91	11.68	0.43	18.40	0.31	0.00	0.01	99.64	38.14	42.97	18.90
1557/25	51.41	0.55	1.88	11.58	0.46	18.78	0.29	0.00	0.03	99.83	38.76	42.59	18.65
1557/26	51.17	0.56	2.06	10.28	0.35	19.72	0.34	0.00	0.10	99.48	40.68	42.77	16.55
1557/27	51.83	0.46	1.86	11.25	0.39	19.14	0.36	0.06	0.05	99.89	39.81	41.93	18.26
Merapi 2006 clinopyroxene													
1083/4	52.67	0.43	2.17	8.27	0.57	21.05	0.35	0.00	n.d.	100.91	42.99	43.82	13.18
1083/6	51.80	0.57	2.90	8.89	0.67	20.84	0.40	0.00	n.d.	100.74	43.23	42.38	14.39
1083/7	52.41	0.44	2.45	8.47	0.53	21.27	0.43	0.00	n.d.	101.32	43.20	43.37	13.43
1083/12	52.26	0.38	1.86	8.27	0.58	20.86	0.30	0.01	n.d.	100.04	42.66	44.14	13.20
1083/16	52.72	0.42	2.21	8.12	0.48	21.02	0.355	0.01	n.d.	100.76	43.03	44.00	12.98
1083/17	52.91	0.34	1.78	8.57	0.70	20.81	0.33	0.00	n.d.	100.33	43.15	42.98	13.87
1083/21	52.78	0.39	2.01	8.39	0.57	20.67	0.37	0.05	n.d.	100.52	42.59	43.93	13.49
1083/22	52.38	0.44	2.25	8.24	0.58	21.49	0.27	0.06	n.d.	100.99	43.68	43.24	13.07
1083/24	53.05	0.38	1.83	8.66	0.59	20.78	0.41	0.00	n.d.	101.44	42.03	44.30	13.67
1083/25	52.53	0.51	2.67	8.21	0.39	21.38	0.41	0.00	n.d.	101.18	43.84	43.02	13.14
1083/26	52.58	0.37	2.23	8.97	0.67	21.18	0.4	0.00	n.d.	101.09	43.55	42.05	14.40
1083/27	52.39	0.44	2.16	8.29	0.59	21.11	0.35	0.00	n.d.	100.61	43.21	43.55	13.24
1083/29	53.14	0.39	2.19	8.58	0.56	21.26	0.38	0.02	n.d.	101.60	43.44	42.87	13.68
1083/32	52.64	0.48	2.46	8.76	0.62	20.48	0.38	0.00	n.d.	101.30	41.91	44.10	13.99
1083/33	52.52	0.42	2.73	8.97	0.58	20.78	0.4	0.00	n.d.	101.24	42.92	42.62	14.46
1083/34	52.88	0.42	2.31	8.43	0.46	20.94	0.34	0.01	n.d.	101.15	42.85	43.68	13.47
1083/35	53.07	0.33	2.28	8.16	0.42	21.28	0.37	0.04	n.d.	101.17	43.58	43.37	13.04
1083/36	52.08	0.61	3.13	8.48	0.50	20.60	0.43	0.00	n.d.	100.53	43.19	42.94	13.88
1083/37	53.34	0.35	1.73	8.34	0.52	20.18	0.32	0.00	n.d.	100.31	41.77	44.75	13.47
1083/38	51.91	0.55	2.97	8.92	0.59	20.16	0.32	0.04	n.d.	100.09	42.46	42.87	14.66
1083/39	53.73	0.27	1.60	8.91	0.55	19.27	0.27	0.00	n.d.	100.65	39.68	46.01	14.32
Kelut 2007 clinopyroxene													
1121/4	51.73	0.33	1.63	9.07	0.46	21.04	0.29	0.01	-	99.33	43.22	42.24	14.55
1121/6	51.41	0.38	1.94	9.22	0.46	20.76	0.29	0.01	-	99.17	42.88	42.26	14.86
1121/17	51.78	0.37	1.76	9.42	0.56	20.09	0.26	0.00	0.05	99.53	41.40	43.44	15.15
1121/18	52.00	0.26	1.64	9.24	0.39	20.50	0.29	0.00	0.03	99.13	42.48	42.58	14.94

Mount/grain	SiO_2	TiO_2	Al_2O_3	FeO	MnO	CaO	Na_2O	Cr_2O_3	V_2O_3	Total	Wo	En	Fs
					Weight %							End-members	
1121/28	50.97	0.33	2.06	9.39	0.42	20.56	0.34	0.06	0.03	98.75	42.68	42.11	15.21
1121/29	51.64	0.22	2.15	8.30	0.55	21.20	0.21	0.00	0.02	98.92	44.20	42.29	13.51
1121/30	51.11	0.38	1.90	9.51	0.51	20.05	0.29	0.00	0.07	98.87	41.43	43.24	15.34
1121/31	51.39	0.34	2.10	9.06	0.57	20.86	0.33	0.03	0.03	99.30	43.30	42.02	14.68

Abbreviations: n/a, not applicable; n.d., not determined. Merapi EPMA data are taken from ref.[1].

Table S5 Source data 2. Raw clinopyroxene oxygen isotope data obtained by SIMS. The table is arranged to show data obtained from the oldest sample first (Agung 1963) through to the youngest sample analysed (Kelut 2007). Within each grouping, the data are sorted by grain number. Raw data obtained for augitic reference material NRM-AG-1 are grouped together for each analytical session.

					Stage position		DTFA		
**Mount/grain @spot number**	**$^{18}O/^{16}O$ drift corrected**	**± absolute**	**$\delta^{18}O$ ‰**	**± ‰**	**x**	**y**	**x**	**y**	**IMF (‰) ref. mat.**
Agung 1963 clinopyroxene – 2020 analysis session – "unknowns"									
1557/28@1	0.00202584	0.00000013	4.72	0.12	1147	680	4	-1	n/a
1557/28@2	0.00202600	0.00000012	4.80	0.11	1045	597	5	-1	n/a
1557/28@3	0.00202666	0.00000012	5.13	0.11	960	482	6	1	n/a
1557/28@4	0.00202649	0.00000017	5.04	0.13	821	420	5	0	n/a
1557/28@5	0.00202661	0.00000013	5.11	0.12	719	398	4	0	n/a
1557/28@6	0.00202665	0.00000012	5.13	0.12	584	364	4	1	n/a
1557/29@1	0.00202662	0.00000015	5.11	0.12	-443	716	-1	0	n/a
1557/29@2	0.00202686	0.00000012	5.23	0.11	-534	651	1	1	n/a
1557/29@3	0.00202648	0.00000012	5.04	0.11	-631	587	-1	0	n/a
1557/29@4	0.00202642	0.00000015	5.01	0.12	-747	488	-1	0	n/a
1557/29@5	0.00202662	0.00000016	5.11	0.13	-838	391	-1	0	n/a
1557/29@6	0.00202595	0.00000013	4.78	0.12	-973	477	-1	0	n/a
1557/38@1	0.00202704	0.00000017	5.32	0.13	14	1563	3	0	n/a
1557/38@2	0.00202671	0.00000019	5.15	0.14	-7	1475	2	0	n/a
1557/38@3	0.00202689	0.00000013	5.24	0.12	-135	1443	2	0	n/a
1557/38@4	0.00202669	0.00000013	5.14	0.12	-248	1461	0	0	n/a
1557/38@5	0.00202708	0.00000012	5.34	0.12	-110	1680	1	1	n/a
1557/38@6	0.00202683	0.00000014	5.22	0.12	-226	1636	0	1	n/a
1557/39@1	0.00202625	0.00000014	4.93	0.12	575	1129	2	-2	n/a
1557/39@2	0.00202587	0.00000013	4.74	0.12	452	1165	4	-2	n/a
1557/39@3	0.00202664	0.00000012	5.12	0.12	394	1310	5	1	n/a
1557/39@4	0.00202678	0.00000012	5.19	0.12	462	1548	4	1	n/a
1557/39@5	0.00202690	0.00000016	5.25	0.12	573	1651	3	1	n/a
1557/39@6	0.00202698	0.00000017	5.29	0.13	686	1735	3	1	n/a
1557/40@1	0.00202699	0.00000016	5.29	0.13	999	2579	5	1	n/a
1557/40@2	0.00202671	0.00000014	5.15	0.12	926	2494	6	1	n/a
1557/40@3	0.00202690	0.00000012	5.25	0.11	778	2472	6	3	n/a
1557/40@4	0.00202705	0.00000014	5.32	0.12	617	2512	4	2	n/a
1557/40@5	0.00202698	0.00000012	5.29	0.12	633	2656	4	2	n/a
1557/40@6	0.00202689	0.00000012	5.25	0.12	530	2739	4	3	n/a
1557/41@1	0.00202652	0.00000013	5.06	0.12	29	3112	1	2	n/a
1557/41@2	0.00202634	0.00000022	4.97	0.15	-56	2956	1	2	n/a
1557/41@3	0.00202663	0.00000017	5.11	0.13	-181	2808	2	3	n/a
1557/41@4	0.00202654	0.00000013	5.07	0.12	-337	2722	0	2	n/a
1557/41@5	0.00202612	0.00000013	4.86	0.12	-544	2654	-1	2	n/a
1557/41@6	0.00202635	0.00000012	4.98	0.12	-728	2657	-1	2	n/a
Agung 1963 clinopyroxene – 2020 analysis session – augitic reference material NRM-AG-1									
1557/AG-1@1	0.00202701	0.00000012	5.30	0.11	1112	-2364	6	-5	0.540

Mount/grain @spot number	$^{18}O/^{16}O$ drift corrected	± absolute	$\delta^{18}O$ ‰	± ‰	Stage position x	y	DTFA x	y	IMF (‰) ref. mat.
1557/AG-1@2	0.00202748	0.00000012	5.54	0.11	1052	-2364	6	-5	0.563
1557/AG-1@3	0.00202765	0.00000012	5.62	0.11	992	-2364	7	-5	0.572
1557/AG-1@4	0.00202734	0.00000017	5.47	0.13	932	-2364	7	-5	0.556
1557/AG-1@5	0.00202742	0.00000012	5.51	0.12	872	-2364	8	-5	0.560
1557/AG-1@6	0.00202720	0.00000012	5.39	0.11	812	-2364	7	-5	0.549
1557/AG-1@7	0.00202701	0.00000015	5.30	0.12	752	-2364	9	-3	0.540
1557/AG-1@8	0.00202738	0.00000012	5.48	0.11	692	-2364	9	-3	0.558
1557/AG-1@9	0.00202744	0.00000012	5.52	0.11	632	-2364	7	-4	0.561
1557/AG-1@10	0.00202743	0.00000012	5.51	0.11	572	-2364	7	-4	0.560
1557/AG-1@11	0.00202723	0.00000019	5.41	0.13	512	-2364	7	-4	0.551
1557/AG-1@12	0.00202740	0.00000012	5.49	0.12	452	-2364	6	-4	0.559
1557/AG-1@13	0.00202732	0.00000012	5.46	0.12	392	-2364	6	-3	0.555
1557/AG-1@14	0.00202698	0.00000012	5.29	0.12	332	-2364	6	-3	0.538

Agung 1963 & Batur 1974 clinopyroxene – 2017 analysis session – "unknowns"
Part 1: Agung 1963 clinopyroxene (grain numbers 30 to 46)

1557/30@1	0.00202083	0.00000024	5.14	0.30	-72	2896	-8	-7	n/a
1557/30@2	0.00202156	0.00000016	5.50	0.28	-642	2813	-11	-4	n/a
1557/31@1	0.00202114	0.00000027	5.30	0.30	-1405	2754	-15	-8	n/a
1557/31@2	0.00202127	0.00000020	5.36	0.29	-1355	3025	-16	-7	n/a
1557/32@1	0.00202145	0.00000022	5.45	0.29	-2635	2988	-23	-8	n/a
1557/33@1	0.00202203	0.00000025	5.74	0.30	-3396	3383	-30	-9	n/a
1557/33@2	0.00202170	0.00000014	5.57	0.28	-3383	4110	-30	-8	n/a
1557/34@1	0.00202120	0.00000015	5.32	0.28	-2717	3876	-25	-7	n/a
1557/34@2	0.00202137	0.00000016	5.41	0.28	-2483	4311	-24	-2	n/a
1557/35@1	0.00202140	0.00000022	5.42	0.29	-1704	4198	-18	-5	n/a
1557/35@2	0.00202114	0.00000022	5.30	0.29	-1790	3819	-19	-5	n/a
1557/36@1	0.00202107	0.00000027	5.26	0.30	-779	3876	-12	-1	n/a
1557/36@2	0.00202015	0.00000023	4.80	0.29	9	4184	-6	-4	n/a
1557/37@1	0.00202071	0.00000014	5.08	0.28	349	3706	-4	-6	n/a
1557/37@2	0.00202091	0.00000026	5.18	0.30	349	3766	-3	-5	n/a
1557/37@3	0.00202114	0.00000023	5.30	0.29	387	3838	-3	-4	n/a
1557/37@4	0.00202065	0.00000031	5.05	0.31	447	3928	-2	-5	n/a
1557/37@5	0.00202021	0.00000021	4.83	0.29	493	4011	0	-4	n/a
1557/37@6	0.00202069	0.00000016	5.07	0.28	548	4100	-1	-5	n/a
1557/37@7	0.00202034	0.00000024	4.90	0.29	559	4220	0	-4	n/a
1557/37@8	0.00201990	0.00000020	4.68	0.29	662	4253	1	-4	n/a
1557/37@9	0.00202360	0.00000024	6.52	0.30	717	4333	2	-3	n/a
1557/37@10	0.00202060	0.00000016	5.03	0.28	778	4417	3	-18	n/a
1557/37@11	0.00202046	0.00000026	4.96	0.30	852	4501	4	-19	n/a
1557/37@12	0.00202006	0.00000019	4.76	0.29	923	4581	4	-19	n/a
1557/37@13	0.00202061	0.00000023	5.03	0.29	1012	4618	6	-18	n/a
1557/37@14	0.00202005	0.00000026	4.75	0.30	1098	4661	6	-18	n/a

Mount/grain @spot number	$^{18}O/^{16}O$ drift corrected	± absolute	$\delta^{18}O$ ‰	± ‰	Stage position x	y	DTFA x	y	IMF (‰) ref. mat.
1557/42@1	0.00202025	0.00000024	4.85	0.29	830	5901	9	-11	n/a
1557/42@2	0.00202038	0.00000034	4.92	0.32	815	5799	10	-12	n/a
1557/42@3	0.00202076	0.00000029	5.11	0.31	721	5678	6	-13	n/a
1557/42@4	0.00202072	0.00000018	5.08	0.28	495	5528	4	-14	n/a
1557/42@5	0.00202094	0.00000021	5.19	0.29	341	5359	2	-12	n/a
1557/42@6	0.00202032	0.00000025	4.88	0.30	251	5294	0	-15	n/a
1557/42@7	0.00201970	0.00000025	4.58	0.30	126	5224	-1	-13	n/a
1557/42@8	0.00202049	0.00000025	4.97	0.30	-26	5151	-3	-12	n/a
1557/42@9	0.00201981	0.00000018	4.63	0.28	-137	5143	-4	-13	n/a
1557/42@10	0.00202014	0.00000017	4.80	0.28	-291	5090	-5	-14	n/a
1557/43@1	0.00202034	0.00000015	4.90	0.28	-406	6633	-3	-9	n/a
1557/43@2	0.00202095	0.00000015	5.20	0.28	-357	6517	-3	-8	n/a
1557/43@3	0.00202006	0.00000027	4.76	0.30	-573	6270	-5	-11	n/a
1557/43@4	0.00201934	0.00000024	4.40	0.30	-733	6346	-7	-11	n/a
1557/43@5	0.00202082	0.00000023	5.14	0.29	-897	6397	-8	-10	n/a
1557/44@1	0.00202149	0.00000023	5.47	0.29	-1528	5692	-16	-13	n/a
1557/44@2	0.00202092	0.00000021	5.19	0.29	-1528	5632	-16	-12	n/a
1557/44@3	0.00202070	0.00000024	5.07	0.29	-1588	5572	-18	-15	n/a
1557/44@4	0.00202076	0.00000015	5.10	0.28	-1648	5512	-17	-14	n/a
1557/44@5	0.00202086	0.00000018	5.16	0.28	-1708	5392	-17	-13	n/a
1557/44@6	0.00202093	0.00000032	5.19	0.31	-1768	5272	-18	-15	n/a
1557/44@7	0.00202098	0.00000015	5.21	0.28	-1768	5152	-18	-16	n/a
1557/44@8	0.00202111	0.00000024	5.28	0.29	-1768	5032	-18	-15	n/a
1557/44@9	0.00202133	0.00000015	5.39	0.28	-1768	4912	-18	-17	n/a
1557/44@10	0.00202090	0.00000025	5.18	0.30	-1768	4792	-18	-17	n/a
1557/44@11	0.00202188	0.00000020	5.66	0.29	-1828	4732	-19	-18	n/a
1557/44@12	0.00202120	0.00000027	5.33	0.30	-1888	4672	-19	-18	n/a
1557/44@13	0.00202169	0.00000022	5.57	0.29	-2065	4801	-20	-18	n/a
1557/44@14	0.00202198	0.00000015	5.71	0.28	-2060	4925	-20	-17	n/a
1557/44@15	0.00202109	0.00000026	5.27	0.30	-2049	5059	-19	-16	n/a
1557/45@1	0.00202068	0.00000025	5.06	0.30	-2137	6449	-20	-11	n/a
1557/45@2	0.00202136	0.00000022	5.40	0.29	-2137	6389	-22	-11	n/a
1557/45@3	0.00202163	0.00000017	5.54	0.28	-2197	6329	-24	-11	n/a
1557/45@4	0.00202097	0.00000025	5.21	0.30	-2257	6209	-23	-13	n/a
1557/45@5	0.00202221	0.00000025	5.83	0.30	-2257	6089	-23	-14	n/a
1557/45@6	0.00202154	0.00000032	5.50	0.31	-2317	6089	-25	-15	n/a
1557/45@7	0.00202139	0.00000024	5.42	0.30	-2317	5969	-24	-15	n/a
1557/45@8	0.00202102	0.00000030	5.23	0.31	-2377	5909	-22	-12	n/a
1557/45@9	0.00202058	0.00000016	5.01	0.28	-2377	5849	-24	-15	n/a
1557/45@10	0.00202047	0.00000026	4.96	0.30	-2377	5789	-24	-15	n/a
1557/45@11	0.00202116	0.00000037	5.30	0.33	-2377	5729	-22	-15	n/a
1557/45@12	0.00202109	0.00000028	5.27	0.30	-2377	5669	-24	-16	n/a
1557/45@13	0.00202084	0.00000022	5.15	0.29	-2377	5549	-24	-17	n/a
1557/45@14	0.00202145	0.00000032	5.45	0.31	-2377	5429	-23	-17	n/a
1557/45@15	0.00202131	0.00000031	5.38	0.31	-2557	5429	-23	-16	n/a
1557/45@16	0.00202211	0.00000031	5.78	0.31	-2557	5549	-24	-15	n/a
1557/45@17	0.00202147	0.00000030	5.46	0.31	-2557	5669	-24	-15	n/a

Mount/grain @spot number	$^{18}O/^{16}O$ drift corrected	± absolute	$\delta^{18}O$ ‰	± ‰	Stage position x	y	DTFA x	y	IMF (‰) ref. mat.
1557/45@18	0.00202204	0.00000033	5.74	0.32	-2557	5789	-25	-15	n/a
1557/45@19	0.00202132	0.00000030	5.38	0.31	-2557	5909	-25	-16	n/a
1557/45@20	0.00202161	0.00000017	5.53	0.28	-2557	6029	-25	-16	n/a
1557/46@1	0.00202151	0.00000021	5.48	0.29	-3009	5308	-28	-18	n/a
1557/46@2	0.00202104	0.00000019	5.24	0.29	-3069	5248	-29	-19	n/a
1557/46@3	0.00202149	0.00000021	5.47	0.29	-3069	5188	-29	-19	n/a
1557/46@4	0.00202195	0.00000028	5.70	0.30	-3069	5068	-28	-17	n/a
1557/46@5	0.00202155	0.00000025	5.50	0.30	-3069	4948	-28	-19	n/a
1557/46@6	0.00202281	0.00000020	6.12	0.29	-3069	4708	-28	-18	n/a
1557/46@7	0.00202308	0.00000036	6.26	0.32	-3369	4708	-31	-21	n/a
1557/46@8	0.00202272	0.00000034	6.08	0.32	-3369	4768	-31	-21	n/a
1557/46@9	0.00202226	0.00000032	5.85	0.31	-3369	4888	-30	-18	n/a
1557/46@10	0.00202154	0.00000019	5.49	0.29	-3309	4948	-30	-20	n/a
1557/46@11	0.00202103	0.00000018	5.24	0.29	-3252	5043	-29	-19	n/a
1557/46@12	0.00202119	0.00000022	5.32	0.29	-3181	5112	-30	-20	n/a
1557/46@13	0.00202142	0.00000024	5.43	0.30	-3092	5183	-29	-20	n/a
Agung 1963 & Batur 1974 clinopyroxene – 2017 analysis session – "unknowns"									
Part 2: Batur 1974 clinopyroxene (grain numbers 6 to 27)									
1557/6@1	0.00202089	0.00000032	5.17	0.31	1397	-6791	-23	-24	n/a
1557/6@2	0.00201983	0.00000030	4.65	0.31	1391	-6535	-24	-26	n/a
1557/6@3	0.00201943	0.00000037	4.44	0.33	1049	-6973	5	-30	n/a
1557/6@4	0.00202457	0.00000028	7.00	0.30	896	-6931	1	-31	n/a
1557/7@1	0.00202021	0.00000029	4.83	0.31	-210	-6785	-44	-27	n/a
1557/7@2	0.00202051	0.00000025	4.98	0.30	387	-6453	-38	-27	n/a
1557/7@3	0.00202229	0.00000028	5.86	0.30	623	-6431	-5	-28	n/a
1557/7@4	0.00202156	0.00000021	5.50	0.29	623	-6507	-5	-30	n/a
1557/12@3	0.00202087	0.00000028	5.16	0.30	-2498	-5455	-38	-25	n/a
1557/12@4	0.00202184	0.00000034	5.64	0.32	-2498	-5515	-38	-22	n/a
1557/12@5	0.00202093	0.00000023	5.19	0.29	-2558	-5515	-39	-24	n/a
1557/12@6	0.00202114	0.00000027	5.29	0.30	-2558	-5575	-37	-23	n/a
1557/12@7	0.00202151	0.00000016	5.48	0.28	-2577	-5717	-40	-24	n/a
1557/12@8	0.00202235	0.00000029	5.90	0.31	-2637	-5836	-42	-26	n/a
1557/12@9	0.00202128	0.00000031	5.36	0.31	-2690	-5902	-42	-23	n/a
1557/12@10	0.00202103	0.00000024	5.24	0.29	-2539	-6062	-41	-24	n/a
1557/13@1	0.00202116	0.00000024	5.31	0.29	-1320	-6124	-29	-15	n/a
1557/14@1	0.00202053	0.00000031	4.99	0.31	-192	-5713	-15	-15	n/a
1557/14@2	0.00202107	0.00000028	5.26	0.30	-98	-5928	-15	-14	n/a
1557/15@1	0.00202209	0.00000032	5.76	0.31	445	-5863	-9	-15	n/a
1557/16@1	0.00202271	0.00000031	6.07	0.31	1119	-6071	1	-15	n/a
1557/17@1	0.00202010	0.00000025	4.78	0.30	1903	-6225	15	-12	n/a
1557/17@2	0.00202194	0.00000021	5.69	0.29	2140	-5974	14	-10	n/a

Mount/grain @spot number	$^{18}O/^{16}O$ drift corrected	± absolute	$\delta^{18}O$ ‰	± ‰	Stage position x	y	DTFA x	y	IMF (‰) ref. mat.
1557/18@1	0.00202040	0.00000020	4.93	0.29	3073	-5974	29	-6	n/a
1557/19@1	0.00201961	0.00000024	4.53	0.29	2942	-5156	23	-8	n/a
1557/20@2	0.00202044	0.00000017	4.95	0.28	2132	-5237	11	-11	n/a
1557/20@2	0.00202048	0.00000014	4.97	0.28	2317	-5170	12	-10	n/a
1557/21@1	0.00202111	0.00000018	5.28	0.29	1794	-5294	5	-9	n/a
1557/22@1	0.00202142	0.00000025	5.43	0.30	318	-4923	-12	-11	n/a
1557/22@2	0.00201967	0.00000019	4.56	0.29	555	-4993	-9	-14	n/a
1557/23@1	0.00201950	0.00000022	4.48	0.29	-688	-5545	-17	-15	n/a
1557/24@1	0.00202092	0.00000019	5.18	0.29	-626	-4459	-21	-13	n/a
1557/24@2	0.00201989	0.00000019	4.67	0.29	-1050	-4769	-25	-11	n/a
1557/25@1	0.00202237	0.00000028	5.91	0.30	-1868	-5311	-32	-14	n/a
1557/26@1	0.00202220	0.00000019	5.82	0.29	-2196	-4667	-33	-13	n/a
1557/27@1	0.00202217	0.00000021	5.81	0.29	-2962	-4923	-40	-9	n/a
Agung 1963 & Batur 1974 clinopyroxene – 2017 analysis session – augitic reference material NRM-AG-1									
1557/AG-1@2	0.00202164	0.00000020	5.54	0.29	705	-1453	-38	-19	0.273
1557/AG-1@3	0.00202117	0.00000026	5.31	0.30	705	-1503	-40	-21	0.250
1557/AG-1@4	0.00202101	0.00000032	5.23	0.31	655	-1603	-40	-21	0.242
1557/AG-1@5	0.00202139	0.00000015	5.42	0.28	655	-1653	-42	-21	0.261
1557/AG-1@6	0.00202202	0.00000017	5.73	0.28	655	-1703	-42	-22	0.292
1557/AG-1@7	0.00202105	0.00000023	5.25	0.29	655	-1753	-42	-21	0.244
1557/AG-1@8	0.00202110	0.00000032	5.28	0.31	655	-1803	-42	-22	0.247
1557/AG-1@9	0.00202122	0.00000021	5.34	0.29	655	-1853	-10	-11	0.253
1557/AG-1@10	0.00202232	0.00000027	5.88	0.30	655	-1903	-13	-11	0.307
1557/AG-1@11	0.00202076	0.00000029	5.11	0.31	545	-1843	-13	-10	0.230
1557/AG-1@12	0.00202049	0.00000032	4.97	0.31	545	-1783	-11	-9	0.217
1557/AG-1@13	0.00202179	0.00000020	5.62	0.29	545	-1723	-14	-10	0.281
1557/AG-1@14	0.00202147	0.00000017	5.46	0.28	545	-1663	-12	-10	0.265
1557/AG-1@15	0.00202204	0.00000018	5.74	0.28	545	-1603	-13	-9	0.293
1557/AG-1@16	0.00202087	0.00000016	5.16	0.28	545	-1543	-12	-9	0.235
1557/AG-1@17	0.00202147	0.00000026	5.46	0.30	485	-1543	-14	-9	0.265
1557/AG-1@18	0.00202137	0.00000028	5.41	0.30	485	-1603	-14	-9	0.260
1557/AG-1@19	0.00202082	0.00000019	5.13	0.29	485	-1723	-14	-9	0.233
1557/AG-1@20	0.00202097	0.00000029	5.21	0.31	305	-1783	-15	-10	0.240
1557/AG-1@21	0.00202098	0.00000019	5.22	0.29	485	-1843	-13	-8	0.241
1557/AG-1@22	0.00202182	0.00000025	5.63	0.30	485	-2023	-14	-9	0.283
1557/AG-1@23	0.00202232	0.00000022	5.88	0.29	485	-2083	-14	-8	0.307
1557/AG-1@24	0.00202166	0.00000024	5.55	0.30	485	-2143	-14	-9	0.274
1557/AG-1@25	0.00202077	0.00000015	5.11	0.28	485	-2203	-13	-25	0.230
1557/AG-1@27	0.00202226	0.00000020	5.85	0.29	485	-2323	-14	-25	0.304

Mount/grain @spot number	$^{18}O/^{16}O$ drift corrected	± absolute	$\delta^{18}O$ ‰	± ‰	Stage position x	Stage position y	DTFA x	DTFA y	IMF (‰) ref. mat.
1557/AG-1@28	0.00202114	0.00000024	5.29	0.30	485	-2383	-11	-26	0.249
1557/AG-1@29	0.00202080	0.00000023	5.12	0.29	545	-2383	-13	-26	0.232
1557/AG-1@30	0.00202123	0.00000028	5.34	0.30	545	-2323	-13	-26	0.253
1557/AG-1@31	0.00202176	0.00000021	5.60	0.29	545	-2263	-13	-26	0.280
1557/AG-1@32	0.00202204	0.00000022	5.74	0.29	605	-2263	-12	-26	0.293
1557/AG-1@33	0.00202078	0.00000028	5.12	0.30	605	-2323	-9	-24	0.231
1557/AG-1@34	0.00202141	0.00000015	5.43	0.28	605	-2383	-12	-26	0.262
1557/AG-1@35	0.00202196	0.00000022	5.70	0.29	665	-2383	-10	-25	0.289
1557/AG-1@36	0.00202182	0.00000048	5.63	0.36	665	-2323	-10	-24	0.282
1557/AG-1@37	0.00202202	0.00000019	5.73	0.29	665	-2263	-11	-25	0.292
1557/AG-1@38	0.00202176	0.00000039	5.60	0.33	665	-2203	-11	-25	0.279
1557/AG-1@39	0.00202148	0.00000015	5.46	0.28	725	-2203	-10	-24	0.265
1557/AG-1@40	0.00202123	0.00000027	5.34	0.30	725	-2263	-10	-24	0.253
1557/AG-1@42	0.00202173	0.00000025	5.59	0.30	725	-2383	-10	-25	0.278
1557/AG-1@43	0.00202170	0.00000025	5.57	0.30	725	-2443	-11	-22	0.276
1557/AG-1@44	0.00202147	0.00000031	5.46	0.31	725	-2503	-10	-24	0.265
1557/AG-1@46	0.00202252	0.00000019	5.98	0.29	725	-2623	-11	-25	0.317
1557/AG-1@47	0.00202023	0.00000025	4.84	0.30	665	-2623	-12	-26	0.203
1557/AG-1@48	0.00202105	0.00000019	5.25	0.29	605	-2623	-12	-25	0.244
1557/AG-1@49	0.00202134	0.00000020	5.40	0.29	485	-2623	-13	-26	0.259
1557/AG-1@50	0.00202077	0.00000042	5.11	0.34	485	-2683	-11	-26	0.230
1557/AG-1@51	0.00202087	0.00000024	5.16	0.30	545	-2683	-12	-24	0.235
1557/AG-1@52	0.00202105	0.00000023	5.25	0.29	605	-2683	-12	-26	0.244
1557/AG-1@53	0.00202222	0.00000044	5.83	0.35	665	-2683	-11	-26	0.302
1557/AG-1@54	0.00202207	0.00000027	5.76	0.30	605	-2743	-12	-27	0.295
1557/AG-1@55	0.00202099	0.00000023	5.22	0.29	545	-2743	-13	-26	0.241
1557/AG-1@56	0.00202050	0.00000045	4.98	0.35	485	-2743	-14	-27	0.217
1557/AG-1@57	0.00202181	0.00000021	5.63	0.29	485	-2803	-13	-26	0.282
1557/AG-1@58	0.00202220	0.00000018	5.82	0.28	545	-2803	-13	-27	0.301
1557/AG-1@59	0.00202037	0.00000017	4.91	0.28	425	-2803	-15	-27	0.210
1557/AG-1@60	0.00202126	0.00000019	5.35	0.29	365	-2803	-15	-26	0.254
1557/AG-1@61	0.00202190	0.00000026	5.67	0.30	305	-2803	-15	-26	0.286
1557/AG-1@62	0.00202213	0.00000031	5.79	0.31	305	-2743	-15	-26	0.298
1557/AG-1@63	0.00202213	0.00000019	5.79	0.29	365	-2743	-15	-27	0.298
1557/AG-1@64	0.00202187	0.00000040	5.66	0.34	425	-2743	-15	-26	0.285
1557/AG-1@65	0.00202139	0.00000029	5.42	0.31	425	-2683	-15	-26	0.261
1557/AG-1@66	0.00202094	0.00000029	5.20	0.31	425	-2623	-15	-26	0.239
1557/AG-1@67	0.00202120	0.00000026	5.32	0.30	365	-2623	-15	-27	0.251
1557/AG-1@68	0.00202126	0.00000028	5.35	0.30	305	-2623	-16	-27	0.254
1557/AG-1@69	0.00202166	0.00000022	5.56	0.29	305	-2563	-15	-27	0.275
1557/AG-1@70	0.00202140	0.00000025	5.42	0.30	365	-2563	-14	-27	0.261

Merapi 2006 clinopyroxene – 2014 analysis session – "unknowns"

1083/4@1	0.00201710	0.00000029	5.33	0.20	5798	2112	52	18	n/a
1083/4@2	0.00201676	0.00000016	5.16	0.17	5636	1915	50	12	n/a
1083/6@1	0.00201690	0.00000018	5.23	0.17	6590	684	58	3	n/a
1083/6@2	0.00201715	0.00000025	5.36	0.19	6666	588	57	3	n/a
1083/6@3	0.00202088	0.00000027	7.21	0.20	6729	533	57	-3	n/a
1083/6@4	0.00201807	0.00000038	5.81	0.24	6739	422	56	-5	n/a

Mount/grain @spot number	$^{18}O/^{16}O$ drift corrected	± absolute	$\delta^{18}O$ ‰	± ‰	Stage position x	Stage position y	DTFA x	DTFA y	IMF (‰) ref. mat.
1083/6@5	0.00201773	0.00000023	5.65	0.18	6684	330	56	-5	n/a
1083/6@6	0.00201658	0.00000019	5.07	0.17	6605	246	54	9	n/a
1083/6@7	0.00201644	0.00000023	5.00	0.19	6605	372	53	11	n/a
1083/6@8	0.00201611	0.00000019	4.84	0.17	6509	432	56	12	n/a
1083/6@9	0.00201613	0.00000027	4.85	0.20	6436	476	53	12	n/a
1083/6@10	0.00201625	0.00000018	4.91	0.17	6385	525	53	12	n/a
1083/6@11	0.00201708	0.00000015	5.32	0.16	6331	581	52	13	n/a
1083/7@1	0.00201678	0.00000030	5.17	0.21	6405	-150	50	11	n/a
1083/7@2	0.00201706	0.00000019	5.31	0.17	6477	-260	49	11	n/a
1083/7@3	0.00201616	0.00000018	4.86	0.17	6876	-113	48	13	n/a
1083/12@1	0.00201829	0.00000021	5.93	0.18	4321	-3815	57	-6	n/a
1083/12@2	0.00201730	0.00000013	5.43	0.16	4395	-3926	58	-6	n/a
1083/12@3	0.00201776	0.00000020	5.66	0.18	4594	-4153	58	-7	n/a
1083/12@4	0.00201798	0.00000028	5.77	0.20	4638	-4208	59	-6	n/a
1083/12@5	0.00201804	0.00000019	5.80	0.17	4701	-4279	59	-6	n/a
1083/12@6	0.00201742	0.00000027	5.49	0.20	4668	-4069	56	-7	n/a
1083/12@7	0.00201684	0.00000020	5.20	0.18	4772	-3973	54	-4	n/a
1083/12@8	0.00201720	0.00000016	5.38	0.17	4939	-3820	50	-2	n/a
1083/12@9	0.00201812	0.00000022	5.84	0.18	5027	-3784	47	-3	n/a
1083/12@10	0.00201667	0.00000020	5.12	0.17	5081	-3697	47	-3	n/a
1083/16@1	0.00201830	0.00000024	5.93	0.19	-1484	-6469	23	-10	n/a
1083/16@2	0.00201823	0.00000021	5.90	0.18	-1394	-6618	24	-13	n/a
1083/17@1	0.00201819	0.00000034	5.87	0.22	-1861	-5716	15	-2	n/a
1083/17@2	0.00201794	0.00000023	5.75	0.18	-1295	-5898	18	7	n/a
1083/21@1	0.00201755	0.00000012	5.56	0.16	-2660	936	11	3	n/a
1083/21@2	0.00201841	0.00000015	5.99	0.16	-2699	935	13	3	n/a
1083/21@3	0.00201800	0.00000012	5.78	0.16	-2739	936	14	3	n/a
1083/21@4	0.00201839	0.00000016	5.97	0.16	-2790	935	17	4	n/a
1083/21@5	0.00201836	0.00000012	5.96	0.16	-2848	938	19	4	n/a
1083/21@6	0.00201809	0.00000020	5.82	0.17	-2795	866	16	5	n/a
1083/21@7	0.00201878	0.00000012	6.17	0.16	-2798	761	16	6	n/a
1083/21@8	0.00201872	0.00000018	6.14	0.17	-2798	660	17	7	n/a
1083/21@9	0.00201943	0.00000027	6.50	0.20	-2795	523	20	6	n/a
1083/21@10	0.00201930	0.00000017	6.43	0.17	-2797	439	20	5	n/a
1083/21@11	0.00201947	0.00000016	6.51	0.16	-2798	327	22	4	n/a
1083/22@1	0.00202133	0.00000024	7.44	0.19	-3673	2214	27	24	n/a
1083/22@2	0.00201997	0.00000017	6.76	0.17	-3312	1983	17	21	n/a
1083/24@1	0.00201797	0.00000037	5.77	0.24	2118	3958	32	25	n/a
1083/24@2	0.00201718	0.00000032	5.37	0.22	2128	3841	32	23	n/a
1083/24@3	0.00201822	0.00000020	5.89	0.18	1973	3675	30	21	n/a
1083/24@4	0.00201678	0.00000017	5.17	0.17	2048	3572	27	19	n/a
1083/25@1	0.00201792	0.00000031	5.74	0.21	4494	3529	55	23	n/a
1083/25@2	0.00201739	0.00000027	5.48	0.20	4649	3309	49	19	n/a

Mount/grain @spot number	$^{18}O/^{16}O$ drift corrected	± absolute	$\delta^{18}O$ ‰	± ‰	Stage position x	y	DTFA x	y	IMF (‰) ref. mat.
1083/25@3	0.00201745	0.00000024	5.50	0.19	4596	3094	45	16	n/a
1083/26@1	0.00201865	0.00000027	6.10	0.20	4107	1347	48	20	n/a
1083/26@2	0.00201723	0.00000019	5.40	0.17	4052	1135	45	18	n/a
1083/26@3	0.00201792	0.00000020	5.74	0.18	3864	1094	43	19	n/a
1083/26@4	0.00201763	0.00000018	5.59	0.17	3645	1092	42	21	n/a
1083/26@5	0.00201770	0.00000022	5.63	0.18	3414	1016	42	19	n/a
1083/27@1	0.00201701	0.00000018	5.28	0.17	4456	2380	45	20	n/a
1083/27@2	0.00201733	0.00000020	5.45	0.17	4292	1954	48	14	n/a
1083/27@3	0.00201752	0.00000019	5.54	0.17	4508	2087	44	17	n/a
1083/27@4	0.00201654	0.00000026	5.05	0.20	4845	2040	45	18	n/a
1083/29@1	0.00201771	0.00000022	5.64	0.18	5186	908	48	18	n/a
1083/32@1	0.00201813	0.00000016	5.85	0.17	2192	-4893	37	2	n/a
1083/32@2	0.00201739	0.00000028	5.48	0.20	2539	-5065	33	-2	n/a
1083/32@3	0.00201668	0.00000019	5.12	0.17	2596	-5222	28	-2	n/a
1083/33@1	0.00201844	0.00000017	6.00	0.17	935	-4981	40	-7	n/a
1083/34@1	0.00201865	0.00000027	6.11	0.20	468	-5913	30	2	n/a
1083/34@2	0.00201759	0.00000029	5.57	0.21	616	-5924	31	3	n/a
1083/35@1	0.00201731	0.00000029	5.44	0.21	472	-3805	18	-6	n/a
1083/35@2	0.00201776	0.00000015	5.66	0.16	444	-3611	21	0	n/a
1083/35@3	0.00201769	0.00000019	5.63	0.17	357	-3481	21	2	n/a
1083/36@1	0.00201811	0.00000014	5.84	0.16	-762	-3378	19	13	n/a
1083/36@2	0.00201907	0.00000030	6.31	0.21	-589	-3453	20	11	n/a
1083/37@1	0.00201881	0.00000023	6.18	0.19	-1288	-3046	26	-4	n/a
1083/37@2	0.00201881	0.00000024	6.18	0.19	-1253	-3035	25	-4	n/a
1083/37@3	0.00201843	0.00000017	6.00	0.17	-1224	-3035	24	-4	n/a
1083/37@4	0.00201819	0.00000017	5.87	0.17	-1194	-3071	24	-5	n/a
1083/37@5	0.00201860	0.00000016	6.08	0.17	-1169	-3092	22	-4	n/a
1083/37@6	0.00201838	0.00000020	5.97	0.18	-1119	-3148	24	-4	n/a
1083/37@7	0.00201816	0.00000025	5.86	0.19	-1084	-3184	23	-4	n/a
1083/37@8	0.00201776	0.00000027	5.66	0.20	-1057	-3238	23	-6	n/a
1083/37@9	0.00201937	0.00000034	6.46	0.22	-1259	-3505	27	-10	n/a
1083/37@10	0.00201805	0.00000027	5.80	0.20	-1143	-3203	24	-5	n/a
1083/37@11	0.00201848	0.00000022	6.02	0.18	-1186	-3240	23	-6	n/a
1083/37@12	0.00201960	0.00000016	6.58	0.17	-1232	-3284	24	-7	n/a
1083/37@13	0.00201871	0.00000020	6.13	0.18	-1310	-3336	25	-9	n/a
1083/37@14	0.00201862	0.00000027	6.09	0.20	-1362	-3379	26	-8	n/a
1083/38@1	0.00202061	0.00000028	7.08	0.20	-2456	3460	22	31	n/a
1083/38@2	0.00202016	0.00000026	6.86	0.19	-2356	3388	23	29	n/a
1083/38@3	0.00201865	0.00000033	6.11	0.22	-2018	3086	19	24	n/a
1083/39@1	0.00201831	0.00000017	5.94	0.17	68	1103	25	20	n/a

Mount/grain @spot number	$^{18}O/^{16}O$ drift corrected	± absolute	$\delta^{18}O$ ‰	± ‰	Stage position x	Stage position y	DTFA x	DTFA y	IMF (‰) ref. mat.
1083/39@2	0.00201821	0.00000022	5.88	0.18	81	773	22	16	n/a
1083/39@3	0.00201858	0.00000022	6.07	0.18	-115	661	23	17	n/a

Merapi 2006 clinopyroxene – 2014 analysis session – augitic reference material NRM-AG-1

1083/AG-1@1	0.00201762	0.00000012	5.59	0.16	-4972	-498	-23	2	0.065
1083/AG-1@2	0.00201799	0.00000024	5.78	0.19	-4974	-440	-23	3	0.083
1083/AG-1@3	0.00201740	0.00000025	5.48	0.19	-4982	-325	-25	5	0.054
1083/AG-1@4	0.00201729	0.00000014	5.43	0.16	-4995	-213	-24	6	0.049
1083/AG-1@5	0.00201774	0.00000023	5.65	0.19	-4996	-135	-26	6	0.071
1083/AG-1@6	0.00201757	0.00000025	5.57	0.19	-5002	-36	-25	7	0.063
1083/AG-1@7	0.00201757	0.00000031	5.57	0.21	-5003	59	-23	8	0.063
1083/AG-1@8	0.00201734	0.00000016	5.45	0.17	-5007	150	-26	8	0.051
1083/AG-1@9	0.00201746	0.00000024	5.51	0.19	-5070	159	-27	9	0.057
1083/AG-1@10	0.00201700	0.00000022	5.28	0.18	-5072	70	-27	9	0.034
1083/AG-1@11	0.00201741	0.00000016	5.49	0.17	-5080	-15	-26	8	0.055
1083/AG-1@12	0.00201734	0.00000027	5.45	0.20	-5078	-200	-26	7	0.051
1083/AG-1@13	0.00201724	0.00000015	5.40	0.16	-5072	-277	-26	6	0.046
1083/AG-1@14	0.00201749	0.00000012	5.52	0.16	-5077	-362	-25	5	0.058
1083/AG-1@15	0.00201733	0.00000029	5.45	0.21	-5073	-431	-25	3	0.051
1083/AG-1@16	0.00201759	0.00000027	5.58	0.20	-5076	-504	-24	3	0.064
1083/AG-1@17	0.00201779	0.00000013	5.68	0.16	-5142	-511	-25	2	0.073
1083/AG-1@18	0.00201756	0.00000016	5.56	0.17	-5144	-426	-26	2	0.062
1083/AG-1@19	0.00201755	0.00000021	5.55	0.18	-5152	-342	-26	4	0.061
1083/AG-1@20	0.00201742	0.00000020	5.49	0.18	-5151	-274	-27	5	0.055
1083/AG-1@21	0.00201800	0.00000016	5.78	0.17	-5154	-196	-27	6	0.084
1083/AG-1@22	0.00201761	0.00000021	5.58	0.18	-5164	-28	-27	8	0.064
1083/AG-1@23	0.00201799	0.00000023	5.78	0.18	-5163	62	-28	5	0.083
1083/AG-1@25	0.00201715	0.00000021	5.36	0.18	-5162	233	-31	18	0.042
1083/AG-1@26	0.00201740	0.00000015	5.48	0.16	-5162	320	-31	19	0.054
1083/AG-1@27	0.00201720	0.00000021	5.38	0.18	-5159	405	-33	20	0.044
1083/AG-1@28	0.00201721	0.00000022	5.39	0.18	-5082	244	-31	20	0.045
1083/AG-1@29	0.00201834	0.00000014	5.95	0.16	-5090	328	-33	23	0.101
1083/AG-1@30	0.00201783	0.00000022	5.70	0.18	-5243	414	-38	21	0.075
1083/AG-1@31	0.00201779	0.00000017	5.68	0.17	-5243	278	-38	18	0.074
1083/AG-1@32	0.00201767	0.00000015	5.62	0.16	-5239	198	-37	17	0.067
1083/AG-1@33	0.00201753	0.00000014	5.55	0.16	-5229	110	-37	17	0.061
1083/AG-1@34	0.00201735	0.00000026	5.46	0.19	-5234	7	-36	17	0.052
1083/AG-1@35	0.00201766	0.00000022	5.61	0.18	-5223	-86	-38	15	0.067
1083/AG-1@36	0.00201738	0.00000026	5.47	0.20	-5231	-189	-37	14	0.053
1083/AG-1@37	0.00201721	0.00000027	5.39	0.20	-5226	-277	-37	12	0.045
1083/AG-1@38	0.00201720	0.00000024	5.38	0.19	-5222	-347	-37	13	0.044
1083/AG-1@39	0.00201755	0.00000025	5.56	0.19	-5219	-412	-37	11	0.062
1083/AG-1@40	0.00201779	0.00000021	5.68	0.18	-5219	-486	-36	11	0.073
1083/AG-1@41	0.00201807	0.00000018	5.82	0.17	-5216	-543	-35	10	0.087
1083/AG-1@42	0.00201718	0.00000033	5.37	0.22	-5277	-527	-36	11	0.043
1083/AG-1@43	0.00201730	0.00000016	5.43	0.17	-5279	-471	-37	11	0.049
1083/AG-1@44	0.00201700	0.00000015	5.28	0.16	-5274	-389	-38	11	0.034
1083/AG-1@45	0.00201741	0.00000020	5.49	0.18	-5274	-314	-39	11	0.055
1083/AG-1@46	0.00201735	0.00000023	5.46	0.19	-5277	-239	-36	12	0.052
1083/AG-1@47	0.00201769	0.00000028	5.63	0.20	-5271	-140	-39	13	0.069

Mount/grain @spot number	$^{18}O/^{16}O$ drift corrected	± absolute	$\delta^{18}O$ ‰	± ‰	Stage position x	y	DTFA x	y	IMF (‰) ref. mat.
1083/AG-1@48	0.00201718	0.00000029	5.37	0.21	-5271	-45	-40	14	0.043
1083/AG-1@49	0.00201717	0.00000024	5.37	0.19	-5283	50	-41	14	0.043
1083/AG-1@50	0.00201761	0.00000035	5.59	0.23	-5289	144	-41	15	0.065
1083/AG-1@51	0.00201793	0.00000030	5.75	0.21	-5300	236	-40	16	0.081
1083/AG-1@52	0.00201792	0.00000029	5.74	0.20	-5297	329	-40	17	0.080

Kelut 2007 clinopyroxene – 2017 analysis session – "unknowns"

1121/4@1	0.00201911	0.00000017	5.48	0.17	-1514	2988	13	-11	n/a
1121/4@2	0.00201981	0.00000024	5.82	0.18	-1648	3005	11	-11	n/a
1121/4@3	0.00201994	0.00000023	5.89	0.18	-1733	3055	10	-11	n/a
1121/4@4	0.00201997	0.00000012	5.90	0.16	-1792	3075	9	-11	n/a
1121/4@5	0.00202067	0.00000012	6.25	0.16	-1841	3086	8	-11	n/a
1121/6@1	0.00201982	0.00000014	5.83	0.16	-182	4235	34	1	n/a
1121/6@2	0.00201936	0.00000015	5.60	0.16	-222	4145	34	0	n/a
1121/6@3	0.00202049	0.00000012	6.16	0.16	-366	3866	33	-1	n/a
1121/6@4	0.00201917	0.00000019	5.50	0.17	-312	3516	31	-9	n/a
1121/17@1	0.00202069	0.00000018	6.26	0.17	-2088	1054	10	-5	n/a
1121/17@2	0.00201960	0.00000016	5.72	0.16	-2130	1030	3	-14	n/a
1121/17@3	0.00201999	0.00000015	5.91	0.16	-2201	1004	3	-16	n/a
1121/17@4	0.00201970	0.00000024	5.77	0.19	-2242	960	4	-16	n/a
1121/17@5	0.00201976	0.00000013	5.80	0.16	-2351	995	3	-16	n/a
1121/17@6	0.00201953	0.00000013	5.68	0.16	-2391	1063	2	-13	n/a
1121/17@7	0.00202032	0.00000017	6.07	0.17	-2244	1071	3	-15	n/a
1121/17@8	0.00202019	0.00000020	6.01	0.17	-2211	1139	3	-13	n/a
1121/17@9	0.00201907	0.00000012	5.45	0.16	-2171	1186	3	-15	n/a
1121/18@1	0.00201850	0.00000016	5.17	0.16	-1097	644	25	-18	n/a
1121/18@2	0.00201939	0.00000016	5.61	0.16	-1164	690	23	-18	n/a
1121/18@3	0.00201951	0.00000019	5.67	0.17	-1336	903	21	-15	n/a
1121/18@4	0.00202050	0.00000021	6.17	0.18	-824	1040	27	-11	n/a
1121/28@1	0.00201760	0.00000018	4.72	0.17	1414	1773	57	-14	n/a
1121/28@2	0.00201870	0.00000016	5.27	0.16	1367	1823	57	-14	n/a
1121/28@3	0.00201774	0.00000016	4.79	0.16	1285	1812	57	-15	n/a
1121/28@4	0.00201785	0.00000018	4.85	0.17	1205	1834	57	-15	n/a
1121/28@5	0.00201964	0.00000017	5.74	0.17	1120	1953	56	-11	n/a
1121/28@6	0.00201921	0.00000018	5.52	0.17	1036	2014	56	-11	n/a
1121/28@7	0.00201867	0.00000019	5.25	0.17	1208	1504	57	-18	n/a
1121/28@8	0.00201831	0.00000013	5.08	0.16	1116	1538	58	-18	n/a
1121/28@9	0.00201852	0.00000021	5.18	0.18	1096	1627	57	-18	n/a
1121/29@1	0.00201835	0.00000012	5.10	0.16	310	1579	37	-20	n/a
1121/29@2	0.00201925	0.00000020	5.54	0.17	179	1686	37	-18	n/a
1121/29@3	0.00201917	0.00000020	5.50	0.17	-13	1871	36	-16	n/a
1121/29@4	0.00201907	0.00000017	5.45	0.17	-215	1774	35	-16	n/a
1121/30@1	0.00201996	0.00000016	5.90	0.17	-2008	2117	4	-15	n/a
1121/30@2	0.00201883	0.00000015	5.33	0.16	-2138	2150	2	-16	n/a

Mount/grain @spot number	$^{18}O/^{16}O$ drift corrected	± absolute	$\delta^{18}O$ ‰	± ‰	Stage position x	Stage position y	DTFA x	DTFA y	IMF (‰) ref. mat.
1121/30@3	0.00201871	0.00000012	5.28	0.16	-2268	2161	1	-16	n/a
1121/30@4	0.00201927	0.00000016	5.56	0.16	-2391	2228	-1	-15	n/a
1121/30@5	0.00201856	0.00000022	5.20	0.18	-2488	2262	3	-14	n/a
1121/30@6	0.00201902	0.00000014	5.43	0.16	-2463	2327	-1	-15	n/a
1121/31@1	0.00201877	0.00000013	5.31	0.16	-2230	3005	-1	-14	n/a
1121/31@2	0.00201941	0.00000016	5.62	0.16	-2281	3285	-6	-16	n/a
1121/31@3	0.00201991	0.00000020	5.87	0.17	-2231	3406	-5	-13	n/a
1121/31@4	0.00201986	0.00000012	5.85	0.16	-2214	3462	-4	-12	n/a
Kelut 2007 clinopyroxene – 2017 analysis session – augitic reference material NRM-AG-1									
1121/AG-1@1	0.00201922	0.00000014	5.53	0.16	-4345	-1172	-31	-19	0.153
1121/AG-1@2	0.00201888	0.00000023	5.36	0.18	-4342	-1055	-32	-20	0.136
1121/AG-1@4	0.00201910	0.00000015	5.47	0.16	-4326	-874	-31	-19	0.147
1121/AG-1@5	0.00201894	0.00000021	5.39	0.18	-4323	-790	-32	-20	0.140
1121/AG-1@6	0.00201913	0.00000018	5.48	0.17	-4314	-700	-33	-20	0.149
1121/AG-1@8	0.00201942	0.00000012	5.63	0.16	-4302	-516	-33	-20	0.163
1121/AG-1@9	0.00201865	0.00000014	5.25	0.16	-4310	-418	-33	-21	0.125
1121/AG-1@11	0.00201924	0.00000017	5.54	0.17	-4294	-238	-34	-21	0.154
1121/AG-1@12	0.00201861	0.00000016	5.22	0.16	-4294	-168	-33	-21	0.123
1121/AG-1@13	0.00201888	0.00000014	5.36	0.16	-4286	-13	-33	-21	0.136
1121/AG-1@14	0.00201956	0.00000014	5.70	0.16	-4286	57	-33	-20	0.170
1121/AG-1@15	0.00201858	0.00000012	5.21	0.16	-4286	127	-33	-20	0.122
1121/AG-1@16	0.00201934	0.00000020	5.59	0.17	-4272	224	-33	-20	0.159
1121/AG-1@17	0.00201932	0.00000025	5.58	0.19	-4272	294	-33	-20	0.159
1121/AG-1@18	0.00201930	0.00000012	5.57	0.16	-4256	448	-32	-19	0.157
1121/AG-1@19	0.00201868	0.00000013	5.26	0.16	-4256	518	-31	-19	0.127
1121/AG-1@20	0.00201924	0.00000017	5.54	0.17	-4256	588	-32	-19	0.155
1121/AG-1@21	0.00201880	0.00000015	5.32	0.16	-4237	708	-33	-19	0.133
1121/AG-1@22	0.00201927	0.00000022	5.55	0.18	-4237	848	-32	-18	0.156
1121/AG-1@23	0.00201936	0.00000013	5.60	0.16	-4237	918	-33	-18	0.160
1121/AG-1@24	0.00201919	0.00000012	5.51	0.16	-4237	988	-32	-18	0.152
1121/AG-1@25	0.00201900	0.00000018	5.42	0.17	-4237	1058	-32	-17	0.142
1121/AG-1@26	0.00201872	0.00000024	5.28	0.19	-4307	1058	-32	-18	0.129

Abbreviations: IMF, instrumental mass fractionation; ref. mat., reference material; n/a, not applicable. Merapi data are shown for completeness and are from ref.[1].

Supplementary References

1. Deegan, F. M. *et al.* Pyroxene standards for SIMS oxygen isotope analysis and their application to Merapi volcano, Sunda arc, Indonesia. *Chem. Geol.* **447**, 1–10 (2016).
2. Gertisser, R. & Keller, J. Trace element and Sr, Nd, Pb and O isotope variations in Medium-K and High-K volcanic rocks from Merapi volcano, Central Java, Indonesia: Evidence for the involvement of subducted sediments in Sunda Arc magma genesis. *J. Petrol.* **44**, 457–489 (2003).
3. Cooper, K. M., Eiler, J. M., Sims, K. W. W. & Langmuir, C. H. Distribution of recycled crust within the upper mantle: Insights from the oxygen isotope composition of MORB from the Australian-Antarctic discordance. *Geochemistry, Geophys. Geosystems* **10**, (2009).
4. Chadwick, J. P., Troll, V. R., Waight, T. E., van der Zwan, F. M. & Schwarzkopf, L. M. Petrology and geochemistry of igneous inclusions in recent Merapi deposits: A window into the sub-volcanic plumbing system. *Contrib. to Mineral. Petrol.* **165**, 259–282 (2013).
5. Hart, S. R. The DUPAL anomaly: A large-scale isotopic anomaly in the southern hemisphere. *Nature* **309**, 753–756 (1984).
6. Kita, N. T. *et al.* Origin of ureilites inferred from a SIMS oxygen isotopic and trace element study of clasts in the Dar al Gani 319 polymict ureilite. *Geochim. Cosmochim. Acta* **68**, 4213–4235 (2004).
7. Troll, V. R. *et al.* Magmatic differentiation processes at Merapi Volcano: Inclusion petrology and oxygen isotopes. *J. Volcanol. Geotherm. Res.* **261**, 38–49 (2013).
8. Jeffery, A. J. *et al.* The pre-eruptive magma plumbing system of the 2007-2008 dome-forming eruption of Kelut volcano, East Java, Indonesia. *Contrib. to Mineral. Petrol.* **166**, 275–308 (2013).
9. Geiger, H. *et al.* Multi-level magma plumbing at Agung and Batur volcanoes increases risk of hazardous eruptions. *Sci. Rep.* **8**, 1–14 (2018).
10. Bindeman, I. N., Ponomareva, V. V., Bailey, J. C. & Valley, J. W. Volcanic arc of Kamchatka: A province with high-$\delta^{18}O$ magma sources and large-scale $^{18}O/^{16}O$ depletion of the upper crust. *Geochim. Cosmochim. Acta* **68**, 841–865 (2004).
11. Preece, K. Transitions between effusive and explosive activity at Merapi volcano, Indoneisa: a volcanological and petrological study of the 2006 and 2010 eruptions. (University of East Anglia, 2014).
